# STAT5a/b Deficiency Delays, but does not Prevent, Prolactin-Driven Prostate Tumorigenesis in Mice

**DOI:** 10.3390/cancers11070929

**Published:** 2019-07-02

**Authors:** Florence Boutillon, Natascha Pigat, Lucila Sackmann Sala, Edouard Reyes-Gomez, Richard Moriggl, Jacques-Emmanuel Guidotti, Vincent Goffin

**Affiliations:** 1 Institut Necker Enfants Malades, Inserm U1151, 75014 Paris, France; 2 Faculté de Médecine, Université Paris Descartes, 75014 Paris, France; 3 Unité d’Histologie et d’Anatomie Pathologique, Ecole Nationale Vétérinaire d’Alfort, 94704 Maisons-Alfort, France; 4 Laboratoire d’Anatomo-Cytopathologie, BioPôle Alfort, Ecole Nationale Vétérinaire d’Alfort, 94704 Maisons-Alfort, France; 5 U955—IMRB, Inserm, Ecole Nationale Vétérinaire d’Alfort, UPEC, 94704 Maisons-Alfort, France; 6 Institute of Animal Breeding and Genetics, University of Veterinary Medicine Vienna, 1210 Vienna, Austria; 7 Medical University of Vienna, 1090 Vienna, Austria

**Keywords:** STAT5, AKT, ERK1/2, prolactin, androgens, prostate cancer, knockout, escape mechanisms, stem/progenitor cells, cell hierarchy

## Abstract

The canonical prolactin (PRL) Signal Transducer and Activator of Transcription (STAT) 5 pathway has been suggested to contribute to human prostate tumorigenesis via an autocrine/paracrine mechanism. The probasin (Pb)-PRL transgenic mouse models this mechanism by overexpressing PRL specifically in the prostate epithelium leading to strong STAT5 activation in luminal cells. These mice exhibit hypertrophic prostates harboring various pre-neoplastic lesions that aggravate with age and accumulation of castration-resistant stem/progenitor cells. As STAT5 signaling is largely predominant over other classical PRL-triggered pathways in Pb-PRL prostates, we reasoned that Pb-Cre recombinase-driven genetic deletion of a floxed *Stat5a/b* locus should prevent prostate tumorigenesis in so-called Pb-PRL^ΔSTAT5^ mice. Anterior and dorsal prostate lobes displayed the highest *Stat5a/b* deletion efficiency with no overt compensatory activation of other PRLR signaling cascade at 6 months of age; hence the development of tumor hallmarks was markedly reduced. *Stat5a/b* deletion also reversed the accumulation of stem/progenitor cells, indicating that STAT5 signaling regulates prostate epithelial cell hierarchy. Interestingly, ERK1/2 and AKT, but not STAT3 and androgen signaling, emerged as escape mechanisms leading to delayed tumor development in aged Pb-PRL^ΔSTAT5^ mice. Unexpectedly, we found that Pb-PRL prostates spontaneously exhibited age-dependent decline of STAT5 signaling, also to the benefit of AKT and ERK1/2 signaling. As a consequence, both Pb-PRL and Pb-PRL^ΔSTAT5^ mice ultimately displayed similar pathological prostate phenotypes at 18 months of age. This preclinical study provides insight on STAT5-dependent mechanisms of PRL-induced prostate tumorigenesis and alternative pathways bypassing STAT5 signaling down-regulation upon prostate neoplasia progression.

## 1. Introduction

Studies of human prostate cancer specimens support a role for prolactin (PRL) signaling in disease progression and recurrence [1]. Indeed, PRL is expressed in more than half of prostate tumors (including local, locally advanced, and hormone refractory) and over 60% metastases [2,3], and its level of expression in primary tumors is positively associated with high Gleason score (i.e., high disease severity) [2]. Since circulating PRL levels are not correlated to prostate cancer risk [4,5], this suggests that the contribution of PRL signaling to prostate tumorigenesis mainly occurs through an autocrine/paracrine mechanism. 

The canonical signaling pathways activated by the PRL receptor (PRLR) involve Janus kinase 2 (JAK2)/Signal Transducer and Activator of Transcription (STAT) pathway, the extracellular regulated kinase (ERK) 1/2 pathway and the phosphoinositide 3-kinase (PI3K)–AKT pathway [6,7]. In human and rodent prostate, the PRLR preferentially signals via the STAT5 pathway; in fact, activation of the other pathways is not detected [2,8,9]. STAT5 involves two highly homologous proteins referred to as STAT5A (94 kDa) and STAT5B (92 kDa) that are encoded by two distinct genes [10]. In PRL signaling STAT5A and STAT5B are activated by JAK2-mediated phosphorylation of a conserved C-terminal tyrosine upon which they form a parallel dimer that allows more efficient translocation into the nucleus where they activate the transcription of target genes involving many protooncogenes. In various preclinical models of prostate cancer, STAT5A/B (hereafter referred to as STAT5 unless specifically discriminated for gene product) has been shown to be critical for cell survival and proliferation through both androgen-dependent and androgen-independent mechanisms [9,11,12,13,14]. Recently, STAT5 was shown to promote epithelial-to-mesenchymal transition (EMT) and stem-like features in human prostate cancer cells [15], supporting the earlier finding that this cascade promotes metastatic properties of prostate cancer cells [16]. Consistent with these observations, inhibition of STAT5 signaling using a dominant-negative STAT5B mutant inhibited the in vitro growth and invasiveness of cell lines derived from the TRAMP (transgenic adenocarcinoma of the mouse prostate) mouse model [17]. Additionally, pharmacological inhibition of the JAK2/STAT5 cascade using the JAK2 inhibitor AZD1480 blocked the growth of primary androgen-dependent as well as the growth of recurrent castrate-resistant prostate cancer (CRPC) xenografts [18]. However, so far no transgenic mouse model studies exist for complete STAT5 deletion to ultimately test its role in prostate cancer initiation and progression, which we investigated here.

In clinical specimens of human prostate cancer, the *STAT5a/b* gene locus was shown to undergo amplification during prostate cancer progression towards metastatic CRPC [19]. Accordingly, the *STAT5a* gene locus was found to be amplified in up to 20% of metastatic CRPC with the neuroendocrine phenotype [20]. Furthermore, STAT5 was shown to be overexpressed in prostate cancer compared to healthy prostate samples, to be positively correlated with Gleason score and to predict recurrence after prostatectomy [2,19,21,22]. These findings corroborated the results obtained in preclinical models and further supported the relevance to develop therapeutic strategies aimed to inhibit STAT5 signaling in prostate cancer. Small molecule inhibitors were designed to block the docking of the SH2 domain of STAT5 to the critical tyrosine of the receptor-JAK2 complex and these have shown potency to inhibit STAT5 tyrosine phosphorylation, nuclear translocation and transcriptional activity [23]. The lead compound (IST5) induced massive apoptosis of prostate cancer cell lines and explant culture of patient-derived prostate cancer [23]. More recently this inhibitor was shown to sensitize prostate cancer to radiation by inhibiting STAT5-mediated DNA repair via the homologous recombination mechanism [24]. Additional observations supporting the role of PRL/STAT5 signaling in prostate tumorigenesis and cancer progression can be found in various review articles published within the last decade [1,14,25,26,27]. 

In contrast to the human prostate [28], expression of the *Prl* gene is not detected in the mouse prostate. Hence, to decipher the molecular and cellular mechanisms linking the autocrine PRL/STAT5 loop to prostate tumorogenesis, we use here a prostate-specific PRL transgenic mouse model. This model involves expression of rat PRL under the control of the prostate-specific, androgen-regulated probasin (Pb) minimal promoter [29]. At six months of age, Pb-PRL mice exhibit dramatically hypertrophied prostates (all lobes) harboring various pre-neoplastic lesions including prostate-intraepithelial neoplasia (PINs), increased stroma density, and inflammation [1,30,31]. They also displayed distended ducts filled with abundant secretions. All these phenotypes aggravate with age. While our seminal report indicated occasional occurrence of invasive cancer in 20 month-old mice [30], these findings were not consistently found in subsequent studies, suggesting possible interference with the genetic background and/or health/microbiota/immune cell status of transgenics that could drift particularly if animal facilities were relocated, as was the case. 

According to earlier findings mentioned above, STAT5 was the only typical PRLR signaling pathway that was found to be activated in Pb-PRL prostates [30]. STAT5 was highly activated in dorsal and lateral lobe, while the ventral lobe displayed much lower level of activation presumably due to PRLR down-regulation specifically in that lobe [31]. Of interest, we discovered that Pb-PRL prostates displayed altered cell hierarchy. First, the basal/stem cell compartment was found to be markedly amplified in the prostate epithelium [30]. Second, these prostates showed amplification of another primitive cell population that had never been described before as it is very rare in the healthy prostate. This newly-identified epithelial cell pool, called LSC^med^ according to its cell sorting profile (Lin−/Sca-1+/CD49f^med^), combines luminal (Cytokeratin [CK] 8 positive) and stem (Stem-cell antigen-1 positive) phenotypic features and exhibits stem/progenitor properties in functional assays [31,32]. Notably, while the basal/stem cell compartment has been long described to be castrate-resistant in rats [33], we recently reported that the LSC^med^ cell compartment survived castration even better than basal/stem cells in mice [32]. 

The amplification of castrate-resistant, stem/progenitor cells in Pb-PRL prostates further supports a role for PRL signaling in the progression of prostate tumors including escape to androgen deprivation. Of interest, careful analysis of immunostaining data suggested that the emergence of these two primitive cell populations in Pb-PRL tumors may be zonally correlated to elevated STAT5 activation [31]. However, as neither basal/stem nor LSC^med^ cell population exhibit detectable levels of PRLR expression [32], the role of STAT5 signaling in their amplification remains elusive and may involve paracrine mechanisms. In order to elucidate the actual contribution of STAT5 signaling in the various hallmarks of PRL-induced prostate tumorigenesis, we took advantage of previously developed floxed *Stat5a/b* mice [34] to abolish STAT5 expression in the epithelial cells of Pb-PRL mice. As reported below, in the anterior and dorsal prostate lobes in which *Stat5a/b* locus deletion was highly efficient, STAT5 deficiency delayed the occurrence of PRL-induced pathological phenotypes in young mice, but could not prevent prostate tumorigenesis in older animals due to the emergence of alternative signaling pathways. 

## 2. Results

### 2.1. STAT5 Deletion has no Detectable Effect on the Prostate Tissue

The analyses of STAT5 expression in the prostate were performed on 6 month-old animals. As shown by RT-qPCR (Figure S1A) *Stat5a* expression was largely prevalent (~5 fold) over *Stat5b* expression in control mice (STAT5^f/f^), but both genes displayed similar lobe-specific expression patterns with the highest expression in anterior and lateral lobes. While the lobe differences were less marked at the protein level, the same profile was observed by immunoblot using the G2 antibody that cross-reacts with both STAT5 isoforms (Figure S1B,C). In ΔSTAT5 mice the fold-reduction in STAT5 expression was the highest in lateral lobe and the lowest in ventral lobe (Figure S1B,C). The residual expression of STAT5 detected at the mRNA and protein levels in tissue extracts of the different lobes of ΔSTAT5 mouse prostates accounted for the unaltered STAT5 expression in non-epithelial cells and possibly to incomplete *Stat5* deletion in the epithelium especially in the ventral lobe (mosaicism). To address this further, we monitored STAT5 expression by immunohistochemistry (IHC) using the G2 antibody (Figure S1D). In ΔSTAT5 prostates a homogenous reduction of STAT5 immunostaining was observed in the epithelium of all but the ventral lobes compared to STAT5^f/f^ prostates (Figure S1D). Together these analyses indicated down-regulation of STAT5A/B expression in the epithelium of the lateral, dorsal and anterior lobes of ΔSTAT5 mouse prostates. 

Prostates harvested from 6, 12, and 18 months old ΔSTAT5 animals failed to display any macroscopic alterations (e.g., organ atrophy or hypertrophy). Accordingly, the weights of the different lobes (normalized to mouse weight) were unaffected by STAT5 deletion (Figure S2A). Histopathological analyses of prostate sections from these animals were performed blinded by a trained veterinarian pathologist (E.R.-G.). This analysis failed to identify detectable alterations in ΔSTAT5 compared to STAT5^F/F^ prostates regarding prostate architecture and histology (Figure S2B). These findings are consistent with an older study that failed to observe histological defects in dorsal and lateral lobes of *Stat5a*-null mice [35]. In fact, systemic STAT5A deficiency induced local disorganization within acinar epithelium of the ventral lobe only, which could not be confirmed in our study as Pb-Cre4-driven *Stat5* gene ablation was inefficient in that lobe. 

Taken together, these results indicate that Pb-Cre4 recombinase-driven *Stat5* deletion has no major impact on prostate tissue integrity. 

### 2.2. Lobe-Specific Pattern of STAT5 Deletion in Pb-PRL^ΔSTAT5^ Mice

**Figure 1:**
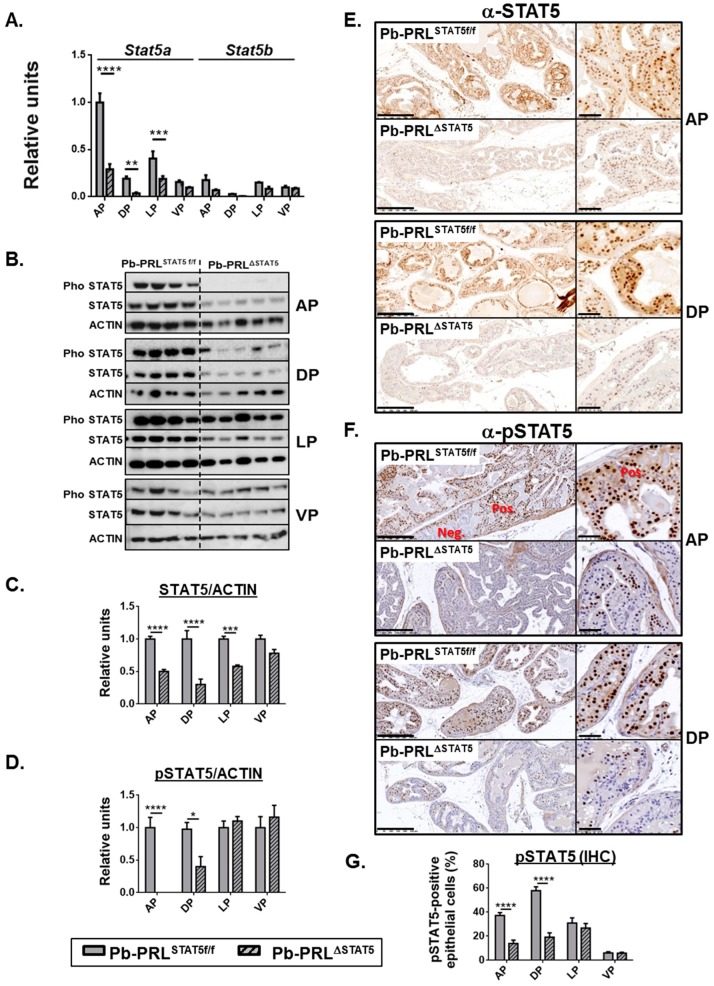
Lobe-specific pattern of STAT5 deletion in 6 month-old Pb-PRL^ΔSTAT5^ mice. (**A**). Lobe-specific expression of *Stat5a* and *Stat5b* mRNA in Pb-PRL^STAT5f/f^ and Pb-PRL^ΔSTAT5^ mice as determined by RT-qPCR is shown. (**B**–**D**). Lobe-specific expression and phosphorylation of STAT5 protein in Pb-PRL^STAT5f/f^ and Pb-PRL^ΔSTAT5^ mice as determined by immunoblot is shown. Quantification of STAT5/ACTIN (C) and pSTAT5/ACTIN (D) was performed by densitometry and is shown as fold change versus Pb-PRL^STAT5f/f^ mice for each lobe. (**E**,**F**). Immunohistochemical analysis of STAT5 protein expression (**E**) and phosphorylation (**F**) in anterior (AP) and dorsal (DP) prostates of Pb-PRL^STAT5f/f^ and Pb-PRL^ΔSTAT5^ mice, as indicated. In panel F, negatively (neg) next to positively (pos) immunostained areas are highlighted. See Figure S3 for the other lobes. (**G**). The percentage of pSTAT5-positive epithelial cells as determined using Calopix software is shown for each lobe. *Statistics*: Stars (* *p* < 0.05, ** *p* < 0.01, *** *p* < 0.001, **** *p* < 0.0001; idem in all figures below) denote significant differences in a repeated-measures two-way ANOVA with Sidak’s multiple comparisons. Size bars: 250 µm in large images and 50 µm in insets.

We next generated Pb-PRL mice harboring *Stat5a/b* gene deletion in the epithelium (hereafter called Pb-PRL^ΔSTAT5^). The analyses of *Stat5a/b* expression were performed on 6 month-old animals. As observed in STAT5^f/f^ mice, *Stat5a* mRNA was predominant over *Stat5b* in Pb-PRL^STAT5f/f^ prostates, with highest levels in anterior and lateral lobes (Figure 1A). In Pb-PRL^ΔSTAT5^ the reduction in *Stat5a/b* mRNA expression was significant in all but the ventral lobes, and more marked in anterior and dorsal (>3-fold) than lateral (<2 fold) lobes (Figure 1A). This pattern was globally confirmed at the protein level using immunoblot and IHC analyses. As shown in Figure 1B (immunoblot) and Figure 1C (quantification), STAT5 protein was efficiently deleted in anterior and dorsal lobes of Pb-PRL^ΔSTAT5^ mice, and the deletion was homogeneous in the epithelium (Figure 1E). In contrast, no significant reduction in STAT5 protein levels were observed in ventral prostate, while deletion in the lateral lobe was intermediate (Figure 1B,C and Figure S3A). 

As earlier reported, STAT5 is massively activated by transgenic PRL overexpressed in Pb-PRL prostates. Therefore tyrosine phosphorylated (p) STAT5 was also monitored by immunoblot (Figure 1B,D) and IHC (Figure 1F,G and Figure S3B) using a validated anti-pSTAT5 antibody [30,31]. Thanks to the contrasted nuclear staining obtained in IHC using the latter antibody (Figure 1F), we could quantify the actual level of activated (nuclear) STAT5 in the luminal epithelium of the four lobes. As expected from above-mentioned studies, the level of STAT5 tyrosine phosphorylation in Pb-PRL^STAT5f/f^ mice was the highest in dorsal lobe and the lowest in ventral lobe (Figure 1G). Also, the pattern of nuclear STAT5 was zonal in the anterior and ventral prostates and more uniformly spread in dorsal and lateral prostates (Figure 1F and Figure S3B). In Pb-PRL^ΔSTAT5^ mice, the significant reduction in STAT5 phosphorylation in anterior and dorsal lobes was assessed by immunoblot (Figure 1B,D) confirming our IHC results of STAT5 down-regulation in epithelial cells (Figure 1F,G). In agreement with the low efficiency of STAT5 deletion in the ventral lobe (Figure 1A,C), no significant reduction of pSTAT5 could be detected in that lobe (Figure 1D,G). Finally, in the lateral lobe, despite of the reduction of STAT5 expression (Figure 1C) there was no significant reduction of STAT5 tyrosine phosphorylation, as determined by blot (Figure 1B) or by IHC (Figure 1G). This is consistent with the fact that the degree of STAT5 phosphorylation in that the lobe is intrinsically moderate in Pb-PRL^STAT5f/f^ mice (Figure 1G), which limits the impact of mild decreased STAT5 expression. 

To confirm that the down-regulation of STAT5 phosphorylation resulted in lower STAT5 signaling, we monitored the levels of expression of Suppressor of Cytokine Signaling (SOCS)/Cytokine-Inducible SH2 Containing Protein (CISH) genes as typical negative regulators and direct targets of JAK/STAT signaling. Based on former reports, SOCS1/2/3 and CISH can be efficiently induced by PRLR/JAK/STAT signaling [7]. SOCS2 was predominantly expressed in the mouse prostate, while SOCS1 was hardly detected (see Figure 6D below for relative expression levels). SOCS2, SOCS3 and CISH were all up-regulated in Pb-PRL^STAT5f/f^ dorsal prostates and significantly down-regulated in Pb-PRL^ΔSTAT5^ confirming lower STAT5 signaling in the latter (Figure S3C). In addition to SOCS genes, we earlier reported that PRLR expression (all isoforms) was down-regulated in Pb-PRL prostates compared to wild-type (WT) littermates [31]; this was confirmed in this study using the dorsal prostate of Pb-PRL^STAT5f/f^ mice (Figure S3D). Notably, normal levels of PRLR expression were restored in Pb-PRL^ΔSTAT5^ prostates suggesting that PRLR expression is negatively regulated by STAT5 signaling (Figure S3D).

In summary, Pb-Cre4-driven *Stat5a/b* deletion was highly efficient to down-regulate STAT5 expression and signaling in the anterior and dorsal prostates but not in the ventral prostate; lateral prostate *Stat5a/b* deletion was intermediate. As described below, these lobe-specific effects were used to delineate the actual involvement of epithelial STAT5 pathway in the various tumor-related phenotypes analyzed. 

### 2.3. STAT5 Deletion Reduces Hallmarks of PRL-Induced Prostate Tumorigenesis at 6 Month of Age

To evaluate the role of STAT5 pathway in PRL-driven prostate phenotypes, we compared the prostates of Pb-PRL^STAT5f/f^ and Pb-PRL^ΔSTAT5^ mice regarding the major hallmarks of early tumorigenesis previously characterized in Pb-PRL prostates [30,31,32,36]. 

#### 2.3.1. Prostate Growth

**Figure 2:**
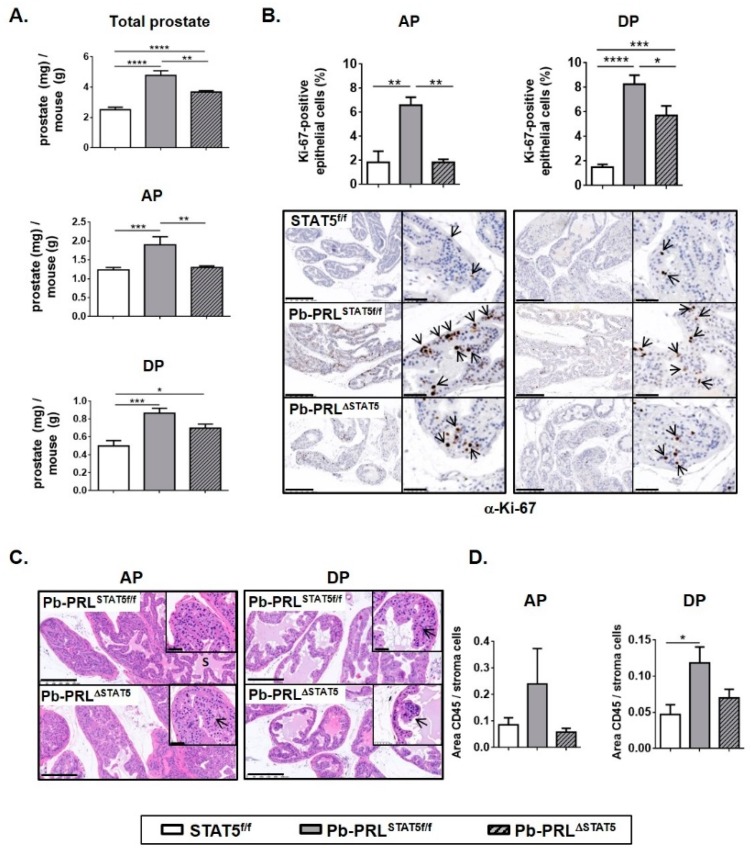
STAT5 deletion reduces hallmarks of early prostate tumorigenesis in 6 month-old Pb-PRL^ΔSTAT5^ mice. (**A**). The weight of total prostate and of anterior (AP) and dorsal (DP) prostate lobes of STAT5^f/f^, Pb-PRL^STAT5f/f^ and Pb-PRL^ΔSTAT5^ mice is expressed as the ratio of prostate tissue weight normalized to the weight of corresponding animal (see Figure S4A for other lobes). (**B**). Cell proliferation in anterior and dorsal lobes was assessed by immunostaining using anti-Ki-67 antibody (hematoxylin counterstaining). Arrows in insets show representative Ki-67 nuclear immunostaining in epithelial cells. The proliferation index (ratio of Ki-67-positive versus total epithelial cells) was quantified using Calopix software (see Figure S4 for other lobes). (**C**). Histological analysis (hematoxylin counterstaining) of anterior and dorsal lobes of Pb-PRL^STAT5f/f^ and Pb-PRL^ΔSTAT5^ mice showing similar hyperplasia in both genotypes (see Figure S4 for other lobes). (**D**). Inflammation was identified using CD45 immunostaining. The degree of inflammation was quantified using Calopix software and is represented as the ratio of CD45+ area versus stroma area. *Statistics*: Stars denote significant differences in a repeated-measures one-way ANOVA with Tukey’s multiple comparisons. Size bars: 250 µm in large images and 50 µm in insets.

According to former reports involving Pb-PRL mice [30], this study confirmed that prostate hypertrophy was detectable from 3 months of age in Pb-PRL^STAT5f/f^ mice compared to control mice. At 6 months of age prostate weight was more than doubled compared to controls (Figure 2A). All lobes significantly contributed to prostate hypertrophy (Figure 2A and Figure S4A). This phenotype was primarily due to cell hyperplasia, as reflected by the higher number of cells obtained in cell sorting (see below Section 2.4) and was further magnified by the increase in prostatic secretions, especially in the anterior prostate. Prostate weight of Pb-PRL^ΔSTAT5^ was significantly reduced compared to Pb-PRL^STAT5f/f^ mice, but remained higher than in control STAT5^f/f^ mice (Figure 2A, top panel). In fact, anterior and dorsal lobes of Pb-PRL^ΔSTAT5^ mice exhibited significant weight loss compared to their counterparts in Pb-PRL^STAT5f/f^ mice (Figure 2A, bottom panels), but this was not the case for ventral and lateral prostates (Figure S4A). These results are in agreement with the lobe-specific pattern of STAT5 signaling down-regulation described above. 

#### 2.3.2. Cell Proliferation

According to their hypertrophic phenotype, all prostate lobes of Pb-PRL mice were previously shown to exhibit increased cell proliferation index (Ki-67 IHC) compared to control mice [36]. Using a computer-assisted image analysis methodology for the quantification of Ki-67-positive cells, this was confirmed in this study involving Pb-PRL^STAT5f/f^ compared to STAT5^f/f^ mice. In agreement with lobe weight reduction and STAT5 signaling down-regulation (see above), a significant reduction in cell proliferation index was observed in the anterior, and to a lesser extent, in the dorsal lobes of Pb-PRL^ΔSTAT5^ mice (Figure 2B), while no change was observed in ventral prostate (Figure S4B). 

#### 2.3.3. Histopathology and Inflammation

At 6 months of age, Pb-PRL displays relatively mild histopathological phenotypes mainly including low grade PINs (early tumorigenesis step) [37,38]. In this study, we failed to find obvious differences between the histopathological features of Pb-PRL^STAT5f/f^ compared to Pb-PRL^ΔSTAT5^ prostates (Figure 2C and Figure S4C). The main phenotypical hallmark was in fact the reduction of the secretory phenotype in the latter, in agreement with the role of PRL signaling in prostate secretory function [39]. 

Inflammatory cell infiltrates are scarce in WT prostates. In contrast, according to previous reports [38,40], Pb-PRL^STAT5f/f^ mouse prostates displayed several foci of CD45+ inflammatory cells especially in the ventral lobe (Figure 2D and Figure S4D). Pb-PRL^ΔSTAT5^ mouse prostates displayed a less marked inflammatory phenotype in the anterior and dorsal lobes (Figure 2D), suggesting a role for luminal STAT5 in this phenotype. In contrast, there was a trend for an increased number of inflammatory cell foci in the two other lobes compared to Pb-PRL^STAT5f/f^ prostates (Figure S4D). 

Taken together, our analyses support a protective role of *Stat5* ablation against the early steps of PRL-triggered prostate tumorigenesis, weakening hyperplasia, lowering cell proliferation, and reducing chronic inflammation. 

### 2.4. STAT5 Deletion Alters Epithelial Cell Hierarchy 

The composition of the prostate epithelium can be determined using cell sorting strategies based on cell surface expression of Stem cell antigen-1 (Sca-1) and Integrin alpha 6 (CD49f). Using these validated markers, luminal and basal (also called LSC^high^) cells can be discriminated [41]. We recently discovered in the mouse prostate a third epithelial cell population that we called LSC^med^ [31]. The basal and LSC^med^ cell populations include stem/progenitor cells and exhibit tumor-initiating capacities when transformed [32,42]. Of interest, in pre-tumoral Pb-PRL prostates both basal and LSC^med^ cell populations are amplified at the expense of the luminal compartment [30,31,32]. 

**Figure 3:**
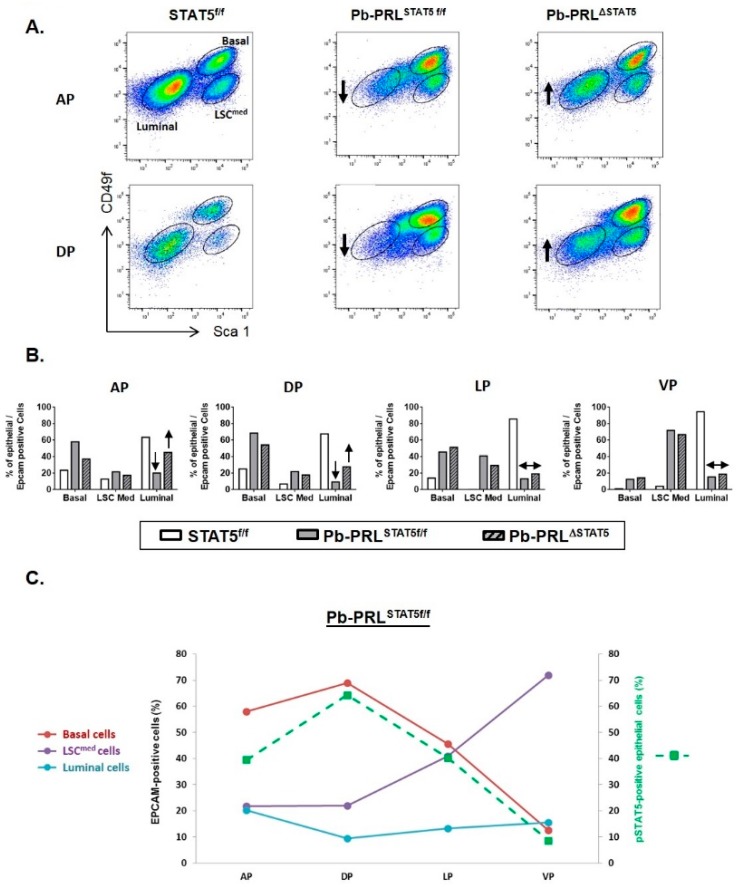
STAT5 deletion alters epithelial cell hierarchy in 6 month-old Pb-PRL^ΔSTAT5^ mice. (**A**). Representative FACS profiles of anterior (AP) and dorsal (DP) prostate lobes from STAT5^f/f^, Pb-PRL^STAT5f/f^ and Pb-PRL^ΔSTAT5^ mice (3–6 animals per genotype). Graphs depict epithelial cells only (gated as Lin-/Epithelial cell adhesion molecule (EPCAM)+), with large gates for percentage analysis. Each FACS profile shows gated epithelial populations: basal/stem (LSC^high^), LSC^med^ and luminal cells. (**B**). Quantification of the three cell populations in the four prostate lobes from the three genotypes is shown. In panels A and B, the arrows indicate the loss of luminal cells in Pb-PRL^STAT5f/f^ versus STAT5^f/f^ mice, and their partial rescue in anterior and dorsal prostate of Pb-PRL^ΔSTAT5^ mice (no similar effect was observed in the two other lobes). (**C**). For each prostate lobe of Pb-PRL^STAT5f/f^ mice, the percentage of the three epithelial cell populations was plotted versus the level of STAT5 phosphorylation, as determined in Figure 1G.

Due to the lobe-specific efficacy of *Stat5* deletion (see Figure 1), we investigated the distribution of these three epithelial cell populations individually in each lobe of the three genotypes of interest using cell sorting. Data in Figure 3A were obtained by pooling 3–6 animals to ensure enough material for analysis. 

In STAT5^f/f^ prostates luminal cells were predominant in all lobes (60% to 90%) and the analysis revealed very low prevalence of basal/LSC^med^ cells in ventral prostate compared to other lobes (Figure 3A,B). In Pb-PRL^STAT5f/f^ prostate, basal and LSC^med^ cells were dramatically amplified in all lobes at the expense of luminal cells that dropped to <20% of the hyperplastic epithelium (Figure 3B). As shown in Figure 3C, the contents in basal and LSC^med^ cells in the various lobes were inversely related (the more basal, the less LSC^med^). Plotting these data against the level of STAT5 phosphorylation in the epithelium of the various lobes of Pb-PRL^STAT5f/f^ mouse prostates, as determined by IHC (Figure 1G) revealed that higher levels of STAT5 activation were associated with higher content in basal cells (Figure 3C). 

Analysis of Pb-PRL^ΔSTAT5^ mouse prostates showed that this enrichment in basal/ LSC^med^ cells was partly reversed upon STAT5 signaling down-regulation. Compared to Pb-PRL^STAT5f/f^ mice the content in luminal cells was partly rescued in the anterior and dorsal lobes of Pb-PRL^ΔSTAT5^ mice at the expense of basal/LSC^med^ cells (Figure 3A,B). No similar effect was observed in lateral and ventral lobes of Pb-PRL^ΔSTAT5^ mice (Figure 3B) in agreement with the unaltered levels of epithelial pSTAT5 compared to Pb-PRL^STAT5f/f^ mice (Figure 1G). 

Taken together, these observations revealed the central role of epithelial STAT5 in the control of epithelial cell hierarchy by local PRL in the mouse prostate. In particular, up-regulation of STAT5 activation led to the accumulation of stem/progenitor cells previously shown to exhibit tumor-initiating capacities when transformed.

### 2.5. STAT5 Deletion does not Promote Alternative PRLR Signaling Cascades 

**Figure 4:**
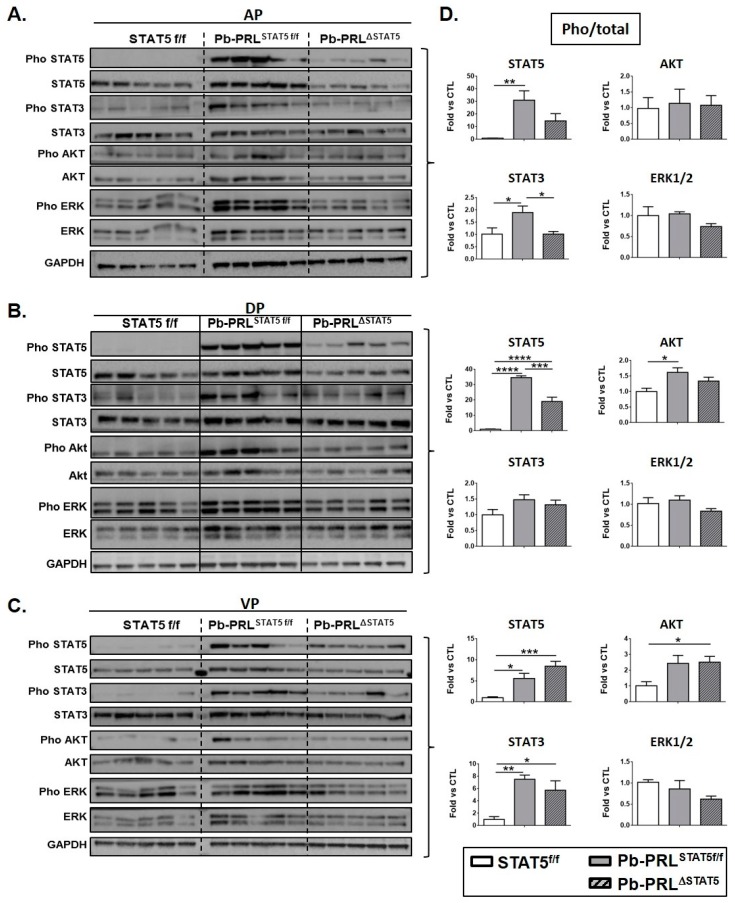
STAT5 deletion does not promote alternative signaling in 6 month-old Pb-PRL^ΔSTAT5^ mice. (**A**–**C**). Canonical PRLR signaling pathways were analyzed by immunoblot in anterior (**A**), dorsal (**B**) and ventral (**C**) prostate lobes from STAT5^f/f^, Pb-PRL^STAT5f/f^ and Pb-PRL^ΔSTAT5^ mice (each lane corresponds to a different mouse). (**D**). The activation of each pathway determined by the ratio of phosphorylated versus total protein (densitometry) is shown as fold-induction versus STAT5^f/f^ samples. *Statistics*: Stars denote significant differences in a repeated-measures one-way ANOVA with Tukey’s multiple comparisons.

In order to investigate whether *Stat5* gene deletion on the Pb-PRL background could promote compensatory PRLR-triggered signaling pathways that may be responsible for the effects reported above in Pb-PRL^ΔSTAT5^ animals, we analyzed the phosphorylation status of STAT5, STAT3, ERK1/2, and AKT in prostates of 6 month old mice of the three genotypes. The immunoblots are shown in Figure 4A (anterior), B (dorsal) and C (ventral) and quantification for each lobe is shown in Figure 4D. 

Virtually no basal phosphorylation could be detected for STAT5 in any lobe of STAT5^f/f^ prostates, which contrasted with STAT3, ERK1/2, and AKT that all showed some background activation. Based on densitometric quantification, STAT5 was strongly activated in dorsal and anterior prostates of Pb-PRL^STAT5f/f^ mice, reaching >30-fold induction compared to control mice. Mild activation (<2 fold) of STAT3 (anterior lobe) and AKT (dorsal lobe) was also observed. In ventral prostate, STAT5 was also activated, but to a much lower degree (~5 fold) than in the other lobes, in agreement with IHC data (Figure S3B). In contrast to the other lobes, the degree of activation of STAT3 in ventral lobe (~7 fold) was in the same range as STAT5 activation. Finally, ERK1/2 was not significantly triggered by PRL in any lobe as shown by unchanged pho/total ERK1/2 ratio (Figure 4D). However, due to slightly enhanced expression of ERK1/2 protein in the anterior lobe of Pb-PRL^STAT5f/f^ prostates compared to STAT5^f/f^ mice (Figure 4A), the tissue content in pERK1/2 in that lobe was significantly increased (*p* < 0.01), and the same trend (not significant) was observed in the other lobes (Figure 4B,C). 

The down-regulation of STAT5 in the anterior (Figure 4A) and dorsal (Figure 4B) lobes of Pb-PRL^ΔSTAT5^ mice did not result in compensatory activation of alternative PRLR signaling pathways. In fact, the pathways that were mildly activated in these lobes in Pb-PRL^STAT5f/f^ prostates, i.e., STAT3 in the anterior lobe and AKT in the dorsal lobe, were back to control levels in Pb-PRL^ΔSTAT5^ prostates (Figure 4D). While this effect suggests that activation of these pathways could be STAT5-dependent, it may also reflect the down-regulation of transgenic PRL protein that was observed in these lobes (Figure S4E). Since the level of PRL mRNA was unaltered (Figure S4F), this suggests that STAT5 signaling may contribute to PRL protein stability. Finally, in agreement with the poor efficiency of *Stat5* deletion in the ventral lobe of Pb-PRL^ΔSTAT5^ mice, PRL expression (Figure S4E) and PRLR signaling pathways (Figure 4C,D) were unchanged in this lobe compared to Pb-PRL^STAT5f/f^ mice.

Taken together, these data indicate that at 6 months of age, STAT5 deletion on the Pb-PRL background does not promote activation of alternative PRLR signaling pathways. 

### 2.6. STAT5 Deletion does not Prevent Prostate Tumor Progression in Aged Pb-PRL Mice 

In order to address the long-term effects of STAT5 signaling deletion on PRL-induced tumor progression, we analyzed Pb-PRL^STAT5f/f^ and Pb-PRL^ΔSTAT5^ mice at 12 and 18 months of age. In the latter, the ventral, lateral, and dorsal lobes were often undistinguishable (hereafter called half prostate) due to massive tissue hypertrophy so that lobe-specific interpretation could not be performed for all investigations. 

**Figure 5:**
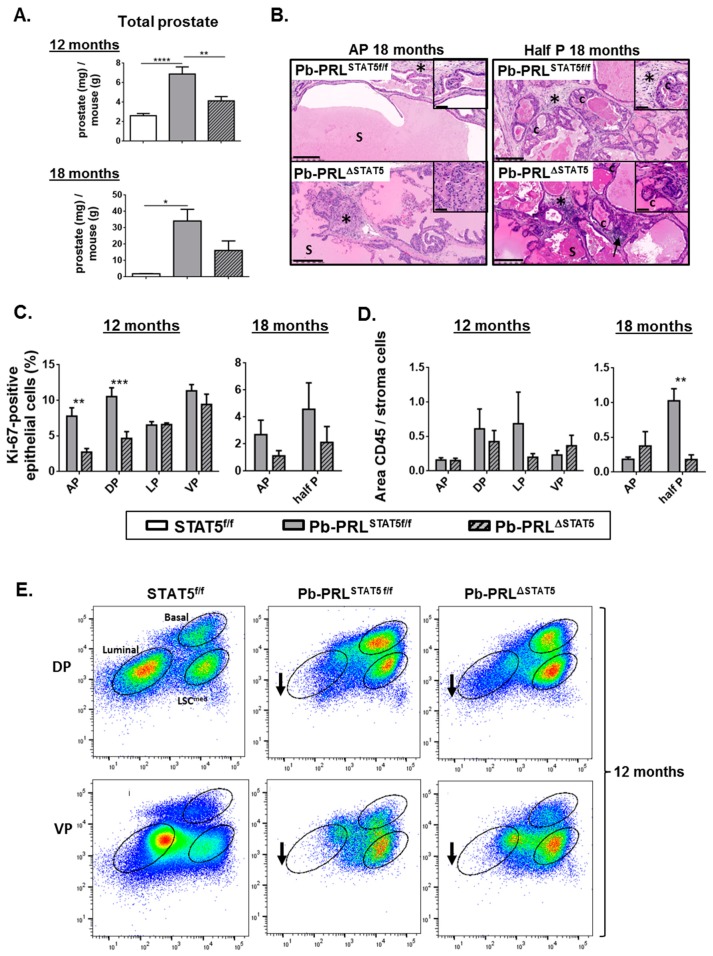
STAT5 deletion does not prevent prostate tumor progression in aged Pb-PRL mice. (**A**). The prostate weight in 12 and 18 month-old STAT5^f/f^, Pb-PRL^STAT5f/f^ and Pb-PRL^ΔSTAT5^ mice is expressed as the ratio of total prostate weight normalized to the weight of corresponding animal (see Figure S5A for data per lobe). (**B**). Histological analysis (hematoxylin counterstaining) of prostate tumors of 18 month-old Pb-PRL^STAT5f/f^ and Pb-PRL^ΔSTAT5^ mice showing similar abnormal histology including PINs, cribriform lesions (“c”), increased stromal density (stars), inflammation (arrows), and dense eosinophilic secretions (S). Sections from the anterior lobe (AP) and from fused dorsal/lateral/ventral lobes are shown. See Figure S5B for 12 month-old animals. (**C**). The proliferation index in prostates from 12 (all lobes) and 18 month old (anterior lobe and total prostate) mice is shown (see Figure 2 for details). (**D**). Inflammation was identified using CD45 immunostaining. The degree of inflammation was quantified using Calopix software and is represented as the ratio of CD45+ area versus stroma area. (**E**). Representative FACS profiles of dorsal (DP) and ventral (VP) prostate lobes from 12 month-old STAT5^f/f^, Pb-PRL^STAT5f/f^ and Pb-PRL^ΔSTAT5^ mice (3–4 animals per genotype; see Figure 3A for details). Arrows show that the loss of luminal cells observed in Pb-PRL^STAT5f/f^ versus STAT5^f/f^ mice was not rescued in Pb-PRL^ΔSTAT5^ mice. *Statistics*: Stars denote significant differences in a repeated-measures one-way ANOVA with Tukey’s multiple comparisons (A) or two-way ANOVA with Sidak’s multiple comparisons (C). Size bars: 250 µm in large images and 50 µm in insets.

The prostate weight of Pb-PRL^STAT5f/f^ was significantly increased at 12 and even more at 18 months of age compared to STAT5^f/f^ mouse prostates (Figure 5A for total prostate and Figure S5A for individual lobes). This weight gain was largely due to the dramatically increased secretory phenotype of Pb-PRL^STAT5f/f^ prostates, especially in the anterior lobe that was extremely swollen/hypertrophic at dissection. This phenotype was confirmed on H&E sections by the presence of abundant and dense eosinophilic secretions in hypertrophied ducts (“S” on Figure 5B and Figure S5B). Compared to Pb-PRL^STAT5f/f^ mice, prostate weight was significantly reduced in Pb-PRL^ΔSTAT5^ mice at 12 months of age (Figure 5A). This effect was correlated to a less marked secretory phenotype and a reduction in cell proliferation that was significant in anterior and dorsal lobes only (Figure 5C). In 18 month-old mice, there was still a trend for reduced prostate weight (Figure 5A) and reduced proliferation index in Pb-PRL^ΔSTAT5^ mice (Figure 5C).

At the histological level, prostates of 12 month old Pb-PRL^STAT5f/f^ mice (Figure 5 and Figure S5B) exhibited various hallmarks of tumorigenesis that further aggravated with age including PINs, cribriform lesions (“c”), increased stromal density (stars) and inflammation (arrows), especially in ventral prostate. While these phenotypes tended to be less pronounced in age-matched Pb-PRL^ΔSTAT5^ mice, they ultimately progressed in the latter so that at 18 months of age the mice of both genotypes became almost undistinguishable irrespective of the lobe (Figure 5B). The inflammatory phenotype was highly heterogeneous at 12 months of age precluding any clear genotype-related difference in any lobe (Figure 5D). There was nevertheless a global trend for lower inflammation in Pb-PRL^ΔSTAT5^ mice, which persisted at 18 months of age (Figure 5D). 

According to the low efficiency of *Stat5* deletion in the ventral lobe, 12 month-old Pb-PRL^ΔSTAT5^ and Pb-PRL^STAT5f/f^ mice displayed very similar cell sorting profiles for this lobe, with accumulation of basal and LSC^med^ cells and low luminal cell content (Figure 5E). Importantly, the dorsal prostates of these animals also displayed very similar profiles enriched in stem/progenitor cells (Figure 5E), indicating that the partial rescue of the luminal cells observed in younger animals upon *Stat5* deletion (Figure 3A,B) was no longer maintained in aged mice. 

Taken together these data indicate that the deletion of STAT5 delayed, but could not prevent the appearance of histopathological hallmarks of PRL-driven prostate tumorigenesis. 

### 2.7. Spontaneous STAT5 Signaling Shutdown and Emergence of AKT and ERK1/2 Signaling in Aged Pb-PRL Mice 

**Figure 6:**
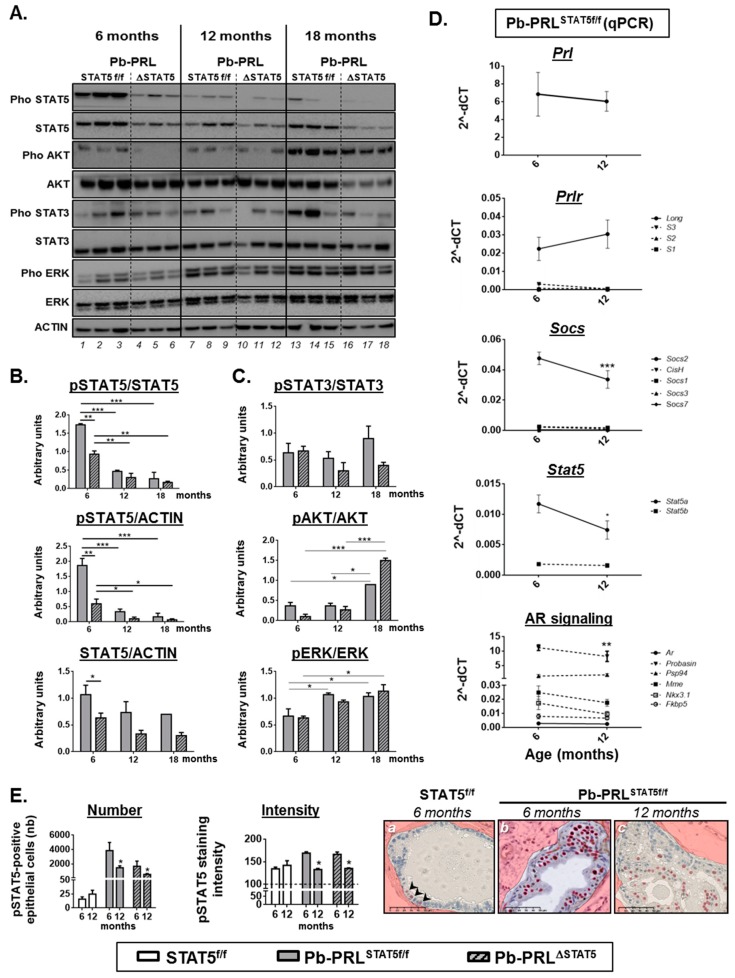
Spontaneous STAT5 signaling shutdown and emergence of AKT and ERK1/2 signaling in aged Pb-PRL mice. (**A**). Canonical PRLR signaling pathways activated in dorsal prostates of Pb-PRL^STAT5f/f^ (lanes 1–3, 7–9, 13–15) and Pb-PRL^ΔSTAT5^ (lanes 4–6, 10–12, 16–18) mice were compared at 6, 12 and 18 months of age as indicated. (**B**). Quantifications of the STAT5 pathway regarding STAT5 activation (pSTAT5/STAT5 ratio), tissue content in activated (pSTAT5/ACTIN) and total (STAT5/ACTIN) STAT5 as determined in panel A. (**C**). The activation of STAT3, AKT and ERK1/2 pathways as shown in panel A was quantified by densitometry as the ratio of phosphorylated versus total protein. (**D**). Age-dependent expression of various actors of the PRLR and AR pathways as determined by RT-qPCR in 6 and 12 month-old Pb-PRL^STAT5f/f^ mice. The dotted line represents low expressed genes of the PRLR pathway (upper panels) and target genes of the AR pathway (bottom panel). See Figure S6 for genotype-dependent expression in 12 month-old mice. (**E**). Comparison of the number of pSTAT5-positive cells and of the intensity of pSTAT5 immunostaining (the horizontal dotted line is the background threshold) in 6 versus 12 month-old STAT5^f/f^, Pb-PRL^STAT5f/f^ and Pb-PRL^ΔSTAT5^ mice. Representative images of low number (arrows)/low intensity (STAT5^f/f^ mice), high number/high intensity (6 month-old Pb-PRL^STAT5f/f^), and high number/low intensity (12 month-old Pb-PRL^STAT5f/f^) are shown (positive cells are red circled). *Statistics*: Stars denote significant differences in a repeated-measures two-way ANOVA with Sidak’s multiple comparisons. Size bars: 50 µm.

To understand further why the protective effect of STAT5 signaling inhibition observed at 6 months of age was progressively lost in old Pb-PRL^ΔSTAT5^ mice, we compared the canonical PRLR signaling pathways in the dorsal prostates of the three genotypes at 6, 12, and 18 months of age (Figure 6A). 

We discovered that in Pb-PRL^STAT5f/f^ mice (lanes 1–3, 7–9 and 13–15 on Figure 6A), the degree of STAT5 activation (pSTAT5/STAT5 ratio) as well as the tissue content in activated STAT5 (pSTAT5/ACTIN) markedly declined between 6 and 12 months of age (Figure 6A,B). We investigated this phenotype further by monitoring the mRNA expression of key players of the PRLR/STAT5 pathway in Pb-PRL^STAT5f/f^ prostate (Figure 6D). There was neither alteration of transgenic *Prl* and *Prlr* expression, nor up-regulation of short PRLR isoforms that have been shown to act as dominant-negative on STAT5 signaling [43]. The lower level of SOCS2 expression at 12- versus 6-months of age assessed that STAT5 signaling down-regulation in the latter was not due to unexpected SOCS overexpression; rather, it was associated with lower STAT5 signaling activity. SOCS7 [44] and nuclear receptor co-repressor 2 (NCOR2, also known as silencing mediator for retinoid or thyroid-hormone receptors, SMRT) [45] are two other negative regulators of STAT5 signaling. None of them were affected at the transcriptional level (Figure 6D and S6A). Otherwise, there was a significant age-related decrease in *Stat5a* mRNA expression (Figure 6D) that was also observed at the protein level in prostate lysates (STAT5/ACTIN ratio), although it did not reach significance, presumably due to unaltered *Stat5* expression in non-epithelial cells (Figure 6B). Comparison of pSTAT5 immunostaining in the epithelium of dorsal prostate of 6- versus 12-month old Pb-PRL^STAT5f/f^ mice revealed that the reduced tissue content in activated STAT5 in older mice was associated with both lower number of pSTAT5-positive cells and, in the latter, reduced intensity of pSTAT5 staining (Figure 6E). Indeed, pSTAT5-positive cells in 12 month old Pb-PRL^STAT5f/f^ mice exhibited faint nuclear staining similar to that observed in the few pSTAT5-positive epithelial cells of healthy prostates (Figure 6E, panels a and c), i.e., markedly lower compared to younger Pb-PRL^STAT5f/f^ animals (Figure 6E, panel b). As a consequence, the level of STAT5 signaling in old Pb-PRL^STAT5f/f^ mice became undistinguishable from that of age-matched Pb-PRL^ΔSTAT5^ mice (see quantifications reported in Figure 6E). Accordingly, most of the targets that were regulated by STAT5 signaling in young animals were not significantly affected by *Stat5* deletion in older mice (Figure S6A). 

Concomitant to the age-dependent decline of STAT5 signaling in Pb-PRL^STAT5f/f^ tumors, we observed a significant increase in ERK1/2 and AKT phosphorylation at 12 and 18 months of age, respectively (Figure 6A,C). Of interest, this phenomenon was also observed in Pb-PRL^ΔSTAT5^ mice, indicating it occurred irrespective of *Stat5* status. There was no clear age-related alteration of STAT3 activation as it remained low in Pb-PRL^ΔSTAT5^ mice and was more variable, but did not significantly increase in PRL^STAT5f/f^ mice (Figure 6A,C). 

Finally, we addressed potential contribution of androgen receptor (AR) signaling to the mechanisms favoring STAT5-independent tumor progression in aged mice of the Pb-PRL background. To that end we investigated the mRNA levels of *Ar* and of several AR target genes [32] in the dorsal prostate of 6 and 12 month old mice. Irrespective of the age, AR signaling was globally lower in Pb-PRL^STAT5f/f^ mice compared to STAT5^f/f^ mice based on the down-regulation of several genes of the AR signature (*Mme*, *Probasin*, *Nkx3.1*; Figure S6B). Additionally, there was also no evidence for age-related increase of AR signaling in Pb-PRL^STAT5f/f^ mice (Figure 6D, bottom panel). Finally, *Stat5* deletion failed to rescue normal AR signaling levels in Pb-PRL^ΔSTAT5^ mice, especially at 12 months of age (Figure S6B). These data clearly indicate that AR signaling up-regulation is not a compensatory mechanism of STAT5 signaling down-regulation in Pb-PRL mice. 

Taken together, our analyses show that STAT5 signaling spontaneously decreases in PRL-driven prostate tumors with aging and our results suggest that alternative compensatory pathways such as AKT and ERK1/2 signaling, but not STAT3 and AR signaling, promote prostate tumor progression upon hyper PRL signaling in the absence of STAT5. 

## 3. Discussion

Reports have provided strong evidence regarding the role of PRL/STAT5 pathway in the progression of human prostate cancer via an autocrine/paracrine mechanism (for reviews, [1,14,25,26,27]. Indeed, this pathway was shown to promote survival, proliferation, EMT, stemness, invasiveness, and DNA repair of/in prostate cancer cells, thereby contributing to prostate cancer progression and resistance to treatment. Several underlying molecular mechanisms have been described, non-exhaustively including up-regulation of PRL expression, STAT5 gene amplification and cooperation with androgen signaling pathway. While some of these biological effects are also observed in Pb-PRL mice (e.g., cell survival, proliferation, and stemness), the latter mice do not develop prostate cancer despite strong STAT5 action [30,31]. This suggested that hyper-activation of STAT5 signaling per se may not act as an oncogene in the prostate, but it could tweak prostate cancer progression in a more proto-oncogenic role driving e.g., enhanced Myc, Bcl2 or D type cyclin family member expressions. However, the promotion of prostate cancer progression with other oncogenic driver pathways identified in the prostate cancer genomic landscape could be complex and STAT5 signaling could be more important in other mutational context [46]. We here present the first genetically-modified mouse model allowing delineation of the actual role of STAT5 signaling in PRL-driven mouse prostate tumorigenesis. 

The lobe-specific pattern of *Stat5* gene deficiency—high in dorsal and anterior lobes, low in ventral lobe of Pb-PRL^ΔSTAT5^ mice—complicated the phenotype/genotype analyses, but at the same time provided the opportunity to assess the functions that are directly controlled by STAT5 signaling by comparing the different lobes within the same prostate sample. These analyses highlighted that epithelial cell survival/proliferation, immune cell infiltration, *Prlr* mRNA expression, and PRL protein stability are tightly dependent on epithelial STAT5 as their regulation (up or down) in Pb-PRL^STAT5f/f^ mice (compared to control mice) was partly or totally reversed in the dorsal and anterior lobes, but not in the ventral lobe of Pb-PRL^ΔSTAT5^ mice. While the STAT5-dependency of prostate epithelial cell survival/proliferation confirms many earlier reports (for a review, see Reference [26]), the molecular mechanisms mediating the epithelial STAT5-dependence of immune cell infiltration remains unknown. While high cell proliferation rate can in some instances be accompanied by increased apoptosis leading to the release of damage-associated molecular pattern (DAMPs) promoting inflammation. However, this is unlikely as Dillner and colleagues provided molecular and immunostaining evidence for reduced apoptosis in Pb-PRL prostates compared to healthy controls [47]. Alternatively, the increased secretory pattern of Pb-PRL prostates may feed the microenvironment with STAT5-induced cytokines/chemokines acting as immune cell chemoattractant. The locally high concentration of PRL may also contribute to inflammation as this hormone has been suggested to mediate inflammation in various pathological contexts, including association with prostate cancer inflammation [48,49]. Finally, the down-regulation of *Prlr* expression by PRL signaling confirmed our previous observation [31] and the reversibility of this phenotype upon *Stat5* ablation (Pb-PRL^ΔSTAT5^ mice) suggested that this negative feedback effect is mediated by STAT5 signaling. 

One of the striking phenotypes harbored by Pb-PRL mice is the amplification of the basal and LSC^med^ cell compartments. This was assessed both by cell sorting and by immunochemistry using cell population-specific phenotypic markers (p63/CK5 for basal and CK4 for LSC^med^ cells) [30,31,32]. The stem/progenitor cells contained in these two cell compartments are highly relevant to cancer progression as they are castrate-resistant and they exhibit tumor-initiating properties when transformed (e.g., by AKT/ERG overexpression or loss of key tumor suppressors in prostate cancer such as PTEN or p53) [32,42,50]. In xenograft experiments, the number of tumor-initiating cells present in the engrafted cell pool is directly correlated to the tumor incidence observed in host mice. In CWR22Rv xenografts, adenovirus-mediated overexpression of a dominant-negative STAT5A/B variant was shown to reduce tumor incidence [11], indicating a reduction in stem-like properties following STAT5 inhibition. This is in agreement with the capacity of STAT5 signaling to increase stem-like phenotypic features and functional properties of human prostate cancer cells in vitro [15]. Thus, the capacity of PRL/STAT5 signaling to induce the amplification of stem/progenitor cells may directly contribute to cancer progression by feeding the prostate tissue with cells able to resist treatments and to regrow a tumor (cancer recurrence). 

**Figure 7:**
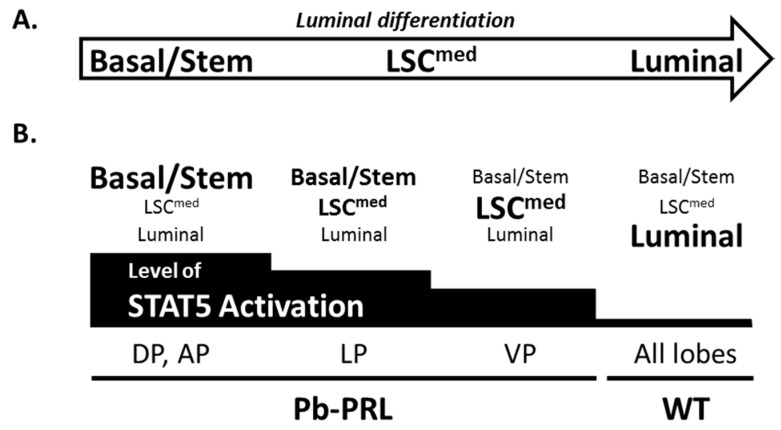
STAT5 signaling prevents luminal differentiation of the prostate epithelium. (**A**). Schematic representation of luminal differentiation placing LSC^med^ cells in-between basal and luminal cells. (**B**). The relative content in the three epithelial cell populations (basal/stem, LSC^med^, luminal) in the various prostate lobes of 6-month old Pb-PRL mice or in WT mice (any lobe), as determined in Figure 3 is symbolized by the size of each population name. Below, the level of STAT5 activation in each lobe as determined in Figure 1 is also schematically represented by the black stair-bar. Based on these measurements, the highest the level of STAT5 activation, the lowest the level of epithelium differentiation was observed (see text for discussion).

In this study, the lobe-specific analyses of pSTAT5 by immunohistochemistry on the one hand, and of the three epithelial cell populations (luminal, basal, LSC^med^) by cell sorting on the other hand, revealed that the prevalence of basal cells was positively correlated to the level of STAT5 activation in the luminal epithelium. The outcome of these analyses is schematized in Figure 7. This finding is in agreement with our former quantitative immunostaining investigations showing that basal cell clusters within the prostates epithelium of Pb-PRL mice are frequently surrounded by luminal cells displaying elevated levels of pSTAT5 [31]. Furthermore, the enrichment in basal/stem cells was partly reversed upon *Stat5* ablation in anterior and dorsal lobes of Pb-PRL^ΔSTAT5^ mice. Another finding of these analyses is that the lobe-specific prevalence of basal/stem cells was inversely correlated to that of LSC^med^ cells. This supports that the latter emanate from the former in the prostate cell hierarchy, as proposed in our detailed study reporting the discovery of LSC^med^ progenitors [31]. Altogether, these observations demonstrate that the prostate cell hierarchy is tightly controlled by epithelial STAT5, which appears to limit luminal differentiation and to favor amplification of the basal/stem compartment (Figure 7). This effect is opposite to what happens in the mammary gland where STAT5A is mandatory for luminal cell differentiation [51]. The mechanism underlying the regulation of prostate cell hierarchy by STAT5 signaling is yet to be elucidated. Transcriptomic profiling of basal and LSC^med^ cells showed that they express only minimal levels of *Prlr* (Ref. [32] and our unpublished qPCR data) therefore their direct regulation by local PRL is unlikely. Paracrine regulation of basal cells by secreted factors downstream of PRL/STAT5 signaling in luminal cells is a possibility that is currently under investigation. As basal and LSC^med^ cells also express detectable levels of *Stat5* (Ref. [32] and our unpublished qPCR data), a cell-autonomous effect may also contribute to the partial reversion of basal cell enrichment in Pb-PRL^ΔSTAT5^ mice. Indeed, although the androgen-regulated *Pb* promoter driving Cre recombinase expression is preferentially active in luminal cells, some reports suggest that it might also be active in basal cells [52]. 

While we earlier identified STAT5 as the main PRLR signaling pathway activated in Pb-PRL mouse prostates [30,31], the fact that pre-neoplastic prostate lesions observed in these animals never (or if at all, only exceptionally) evolve towards malignancy has always seemed in contradiction with the strong experimental and clinical evidence supporting the promoting role of this pathway in human prostate cancer (see Introduction). Human prostate specific PRL signaling could be more important than that of rodent prostates to drive tumorigenesis. Additionally, human prostate cancer is predominant in elder men and the various genetic and environmental risk factors (e.g., smoking, alcohol abuse, etc.) could insufficiently be recapitulated in inbred *C57Bl/6J* transgenics as used here as a study subject. Irrespective, the present work provides a possible explanation for the absence of prostate cancer development in Pb-PRL mice as we show that the prostates of these animals progressively lack STAT5 signaling with age. To address whether continuously elevated STAT5 signaling is actually required for the transformation of pre-neoplastic prostate lesions, it would be interesting to generate mice with enforced expression of a constitutively-active STAT5 variant in the prostate [53]. In conclusion, we show, for the first time, that aging can promote alternative compensatory pathway activations to bypass STAT5 function, but inflammation, cell proliferation and survival as well as prostate epithelium differentiation are significantly affected by PRL-STAT5 signaling.

The mechanism driving age-related decline of STAT5 signaling in the prostates of ageing Pb-PRL^STAT5f/f^ mice remains unclear. No parallel up-regulation of the classical negative regulators of STAT5 signaling (SOCS proteins, short PRLR isoforms) could explain this observation. Although we noticed a decrease in STAT5 expression (mRNA and protein) in the dorsal prostates of 12 month old Pb-PRL^STAT5f/f^ mice, this alone was insufficient to account for the pronounced loss of STAT5 activation (Figure 6B), suggesting that other mechanisms may be involved. In the breast cancer context, it has been shown that increased stiffness/density of the extracellular matrix around the tumor as a result of altered collagen deposition during cancer progression shifted the balance of PRLR signaling from JAK2/STAT5 to other pathways including ERK1/2 and AKT pathways through a Focal adhesion kinase-dependent mechanism [54,55]. Of interest, the pre-neoplastic lesions of Pb-PRL mouse prostates also exhibit strong remodeling of the extracellular matrix compared to controls, including >10 fold-increased expression of various Collagens [47]. This phenotype aggravated with age, concomitant to the decline in STAT5 signaling and up-regulation of AKT and ERK1/2 signaling, therefore it is possible that a similar mechanism as described in breast cancer contributes to the signaling shift observed in the prostates of ageing Pb-PRL^STAT5f/f^ mice. No such up-regulation of AKT or ERK1/2 signaling was observed when STAT5 signaling was genetically impaired in younger Pb-PRL^ΔSTAT5^ animals, further supporting that the progressive up-regulation of alternative signaling pathways in Pb-PRL^STAT5f/f^ mice involves age-related mechanisms that require time to establish. These alternative signaling pathways allowed pre-neoplastic lesions to develop further in a STAT5-independent manner, as reflected by the elevated levels of cell proliferation and inflammation, and by the appearance of various histopathological phenotypes including cribriform lesions in old mice. In fact, these phenotypes were observed irrespective of *Stat5* status. Thus, although *Stat5* deficiency delayed the occurrence of various pre-neoplastic hallmarks, its protective effect was progressively diluted with age to become almost undetectable in 18 month-old animals. Of note, neither androgen nor STAT3 signaling could be identified as a compensatory pathway of age- or genetically-induced down-regulation of STAT5 signaling. There was even a trend for lower STAT3 signaling in *Stat5*-deficient prostates, suggesting some level of cooperation between both pathways. 

Preventing the progression of established prostate cancers is where the therapeutic challenge stands. In agreement, there is currently strong research to develop STAT5 inhibitors that could be used for the treatment of various malignancies, including prostate cancer and myeloid leukemia [23,24,56]. Whatever the mechanisms driving the intrinsic decline of prostatic STAT5 signaling in Pb-PRL mice, the various genotype- and age-related phenomena observed in our study may shed light on possible outcomes of long-term pharmacological inhibition of STAT5 in prostate cancer context. On the good side, as low STAT5 signaling parallels luminal differentiation, pharmacological STAT5 inhibitors are not expected to result in tumor enrichment in stem/progenitor cells, but they can also act on the stromal cells such as immune cells. This effect may distinguish anti-STAT5 treatments from current therapeutic strategies (castration, chemotherapy) which generally kill luminal cells and let stem/progenitor cells unaffected, which is believed to favor tumor recurrence based on the tumor-initiating capacities of the latter. In addition, STAT5 inhibition may also decrease autocrine PRL stability as suggested from our results, which may further contribute to drug efficacy as PRL is presumably the major upstream factor triggering STAT5 pathway in the prostate. On the negative side, our study identified potential escape mechanisms to STAT5-targeted therapeutic strategies. In young Pb-PRL^ΔSTAT5^ mice, which model early stages of STAT5 inhibition, no activation of alternative PRLR signaling pathway could be identified, and cell proliferation was actually reduced. With time, which is modeled by old Pb-PRL^ΔSTAT5^ mice, we observed up-regulation of AKT and ERK1/2 signaling. One might speculate that in a context of pharmacological STAT5 inhibition these pathways may contribute to STAT5-independent prostate cancer progression. It is currently unknown whether AKT and ERK1/2 are triggered by PRL only or whether other paracrine growth factors will contribute. With the aim to design appropriate treatment combination limiting escape mechanisms to STAT5 inhibition, it may be relevant to address this mechanism in future studies. 

## 4. Materials and Methods

### 4.1. Transgenic Mouse Strains

The Pb-PRL mouse colony carrying the rat prolactin transgene driven by the short *probasin* promoter was established on the *C57BL/6J* background (>20 backcrosses) in the Paris laboratory as previously described [30]. Mice carrying floxed *Stat5a/b* alleles (STAT5^f/f^ mice) allowing targeted deletion of the whole *Stat5* locus (encompassing both *Stat5a* and *Stat5b* genes) were originally developed by L. Hennighausen [34]. Animals were obtained from the in-house colony of R. Moriggl (Vienna, Austria) established on a pure *C57Bl/6* genetic background. Pb-Cre mice (C57Bl/6 background) expressing the Cre recombinase under the control of the *short probasin* promoter [57] were purchased from the Frederick National Laboratory for Cancer Research (Frederick, MD, USA). Figure S7 displays the breeding scheme used to generate mice carrying or lacking prostate epithelium-specific deletion of *Stat5a/b* genes on the WT (STAT5^f/f^ and ΔSTAT5 mice) and Pb-PRL (Pb-PRL^STAT5f/f^ and Pb-PRL^Δ^^STAT5^ mice) backgrounds. Note that only males were used to transmit the *Pb-Cre* allele. 

Colonies were housed in controlled conditions, on a 12/12-hour light/dark cycle with normal food and water provided *ad libitum*. Mice were analyzed at 6, 12 and 18 months of age, and prostate were harvested immediately after sacrifice by cervical dislocation. To isolate the prostate, dissection of the urinary tract was performed and left lobes were separately dissected, weighed then rapidly snap frozen while the right-sided half prostate was fixed in paraformaldehyde (PFA) without being further dissected, so that tissue organization was preserved for histological analysis [36]. For all genotypes, we analyzed the four prostate lobes (anterior, dorsal, lateral, ventral) at all ages. Note that at 18 months of age, dorsal, lateral, and ventral lobes were often fused due to tissue hypertrophy. 

Animal experiments were approved by the local ethical committee—Comité d’Ethique en matière d’Expérimentation Animale Paris Descartes (CEEA 34) for animal experimentation (authorization CEEA34.VG.095.12) and performed according to the European guidelines for animal experimentation. 

### 4.2. Prostate Subpopulation Sorting by FACS

The procedures for sorting the three epithelial cell populations based on their Lin/Sca-1/CD49f antigenic profile was previously described [31,32,41]. Cell sorting was performed on a BD FACSAria III (BD Biosciences, San Jose, CA, USA). Sorted cells were collected in DMEM medium, supplemented with 50% FBS, glutamine, and penicillin-streptomycin, or in RA1 Lysis Buffer (Macherey-Nagel, Düren, Germany) to perform RNA extraction as earlier described [32].

### 4.3. Quantitative PCR

Total RNA was isolated from separate prostate lobes using the NucleoSpin^®^ RNA XS (Macherey Nagel, Hoerd, France) according to manufacturer’s instructions. RNA integrity was assessed on Agilent BioAnalyzer (all RNAs scored 7–10). RNA (250 ng) was reverse transcribed using SuperScript™II Reverse transcriptase with the SuperScript™II First-Strand Synthesis System for RT-PCR kit (Invitrogen, CA, USA). The cDNAs were then subjected to real-time PCR amplification using gene-specific primers (0.5 µM final concentration) purchased from IDT DNA (Integrated DNA Technologies, BVBA, Leuven, Belgium; HPLC purification; referred to as Mm.PT in Table S3) or Eurogentec (Liège, Belgium; Oligold quality, sequence given in Table S1). PPIA (Peptidyl Prolyl Isomerase A) that encodes Cyclophilin A that was used as the housekeeping gene in each reaction. Real-time PCR was performed using a Qtower 2.0 (Analytik Jena, Germany). The qPCR reaction contained 2 µL cDNA sample (12.5 ng) and 8 µL mastermix with 1× GoTaq^®^ qPCR Master Mix (Promega, Charbonnières-les-Bains, France) and 0.5 µM primer. The Qtower 2.0 Instrument was used with the following program: Enzyme activation: 95 °C for 2 min; amplification (40 cycles): 95 °C for 15 s, 60 °C for 60 s. Results were generated with the Qtower 2.0 software and were analyzed by the comparative cycle threshold method and presented as fold change in gene expression relative to internal calibrators as mentioned in figures. 

### 4.4. Western Blotting

Freshly dissected prostate lobes were snap frozen in liquid nitrogen and stored at −80 °C until processing as previously described [31]. Protein samples (20–50 µg) were resolved in 4–12% gradient SDS-PAGE in NuPAGE Bis-Tris Precast Gels (Life Technologies, Saint-Aubin, Fance) or 10% SDS-PAGE gels. Proteins were then transferred onto nitrocellulose membranes (BioRad, Marnes-la-coquette, France). Membranes were cut horizontally according to the size of the proteins of interest and stained with primary antibodies as described in Table S1. For band detection, Horseradish Peroxidase (HRP)-coupled secondary anti-rabbit (7074, Cell Signaling, Saint-Quentin-en-Yvelines, France) or anti-mouse (NA931, GE Healthcare Europe, Freiburg, Germany) antibodies were added before Enhanced Chemiluminescent (ECL) substrate (Immobilon Western Chemiluminescent HRP Substrate, Millipore or LumiGLO Reagent, Cell Signaling). Representative parts of blots are shown in the Figures, whole blot membranes are displayed in Figure S8.

### 4.5. Prostate Histopathology

Histopathological diagnosis of prostate sections from all mice was performed in blind by an independent veterinary pathologist (E.R.-G.) trained for mouse prostate analysis [36] following the recommendations of the Mouse Models of Human Cancer Consortium Prostate Pathology Committee and the reference classification of prostate intraepithelial neoplasia (PIN) lesions in genetically-modified animals [58,59]. This qualitative analysis was complemented by quantitative IHC analyses, as described below. 

### 4.6. Immunohistochemistry (IHC) 

All prostate samples were fixed in 4% PFA, paraffin embedded, and underwent heat-induced antigen retrieval in citrate buffer at pH 6. IHC and IF were performed as previously described [31] using antibodies listed in Table S2. Vector Elite ABC HRP kit with DAB substrate (Vector Laboratories, Burlingame, CA, USA) was used for detection of IHC slides, with hematoxylin as counterstain. 

### 4.7. Image Acquisition and Histology Quantifications

Prostate tissue sections (H&E or IHC) were digitally scanned using a NanoZoomer-2.0 RT scanner (Hamamatsu, Photonics, France) coupled to NDP.view2 software analysis beta version U12388-01 (Hamamatsu, Photonics, France). For quantification of immunostaining, computer-assisted analysis of digital (scanned) images was performed using Calopix software (http://www.tribvn.com/). To quantify nuclear immunostaining (pSTAT5, Ki-67) in the epithelium, first the Random Forest Tree for tissue recognition was used to delineate and only include the glandular areas in the analysis. Then, the “morphometry” software was applied to each prostate lobe to discriminate staining-positive (DAB+) versus staining-negative (DAB−) cells. A value of marking intensity (optical density, <100 considered as background) was also provided by the algorithm for both classes of cells. The index of proliferation (Ki-67+) and of STAT5 activation was calculated as the ratio of the number of positive versus total (positive + negative) cells counted. The whole procedure was validated by comparing the results obtained by Calopix-assisted versus manual counting of digital images as previously reported [36,38,60]. For the quantification of CD45 staining, results are expressed as the ratio between the surface of CD45+ cell foci versus the total surface of stroma [36,38,60]. 

### 4.8. Statistics

The specific statistical tests performed are described in the legends for all results reported. In summary, Analysis of Variance (ANOVA) tests were used to evaluate differences among three or more groups. Depending on the number of factors tested, One or Two-way ANOVAs were used. When prostate lobe samples issued from the same mice were compared in the analysis, a repeated-measures test was carried out. Post hoc multiple comparisons were performed with Sidak’s or Tukey’s multiple comparisons. One, two, three or four symbols illustrating significance represent *p* values <0.05; <0.01; <0.001 and <0.0001, respectively. A value of *p* < 0.05 was used as significance cutoff for all tests. Error bars represent S.E.M. All analyses were performed using GraphPad Prism version 6.00 for Windows (GraphPad Software, San Diego, CA, USA).

## 5. Conclusions

This work confirmed STAT5 signaling as a major driver of autocrine PRL-mediated prostate tumorigenesis as genetically-induced STAT5 down-regulation delayed the onset of various PRL-induced tumor hallmarks. The age-related decline in STAT5 signaling observed in Pb-PRL mice harboring intact *Stat5* genes was unexpected, and may explain why malignancies failed to develop despite alternative signaling pathways were switched on and presumably contributed to the progression of pre-neoplastic lesions. *Stat5* genotype-related differences of prostate phenotypes observed in young animals were progressively lost in ageing mice. Together, these findings highlight an important role of PRL/STAT5 in the development of prostate tumors. 

## Supplementary Materials

The following are available online at https://www.mdpi.com/2072-6694/11/7/929/s1, Figure S1. Lobe-specific pattern of STAT5 deletion in 6 month-old STAT5 mice, Figure S2. STAT5 deletion has no detectable effect on the prostate tissue, Figure S3. Lobe-specific pattern of STAT5 deletion of 6 month-old Pb-PRL STAT5 mice (complementary to Figure 1), Figure S4. Lobe-specific effects of STAT5 deletion of 6 month-old Pb-PRL STAT5 mice (complementary to Figure 4), Figure S5. STAT5 deletion does not prevent prostate tumor progression in aging Pb-PRL mice (complementary to Figure 5), Figure S6. Characterization of PRLR and AR signaling pathways in dorsal prostate of 12 month-old STAT5f/f, Pb-PRLSTAT5f/f and Pb-PRL STAT5 mice (complementary to Figure 6), Figure S7. Breeding scheme, Figure S8. Whole Western blot membranes corresponding to representative images shown in main and supplemental Figures, as indicated. Table S1: Antibody references and conditions of use, Table S2: Primers used for qPCR.
