# Supplementary material for: STAT5a/b Deficiency Delays, but does not Prevent, Prolactin-Driven Prostate Tumorigenesis in Mice
Source: Cancers (Basel). 2019 Jul 2;11(7):929. doi: 10.3390/cancers11070929 (PMC6678910; doi:10.3390/cancers11070929)
Supplement: Supplementary file 1 [file cancers-11-00929-s001.pdf]

Supplemental Materials: STAT5a/b Deficiency Delays, but does not Prevent, Prolactin-Driven Prostate Tumorigenesis in Mice

Florence Boutillon, Natascha Pigat, Lucila Sackmann Sala, Edouard Reyes-Gomez, Richard Moriggl, Jacques-Emmanuel Guidotti and Vincent Goffin

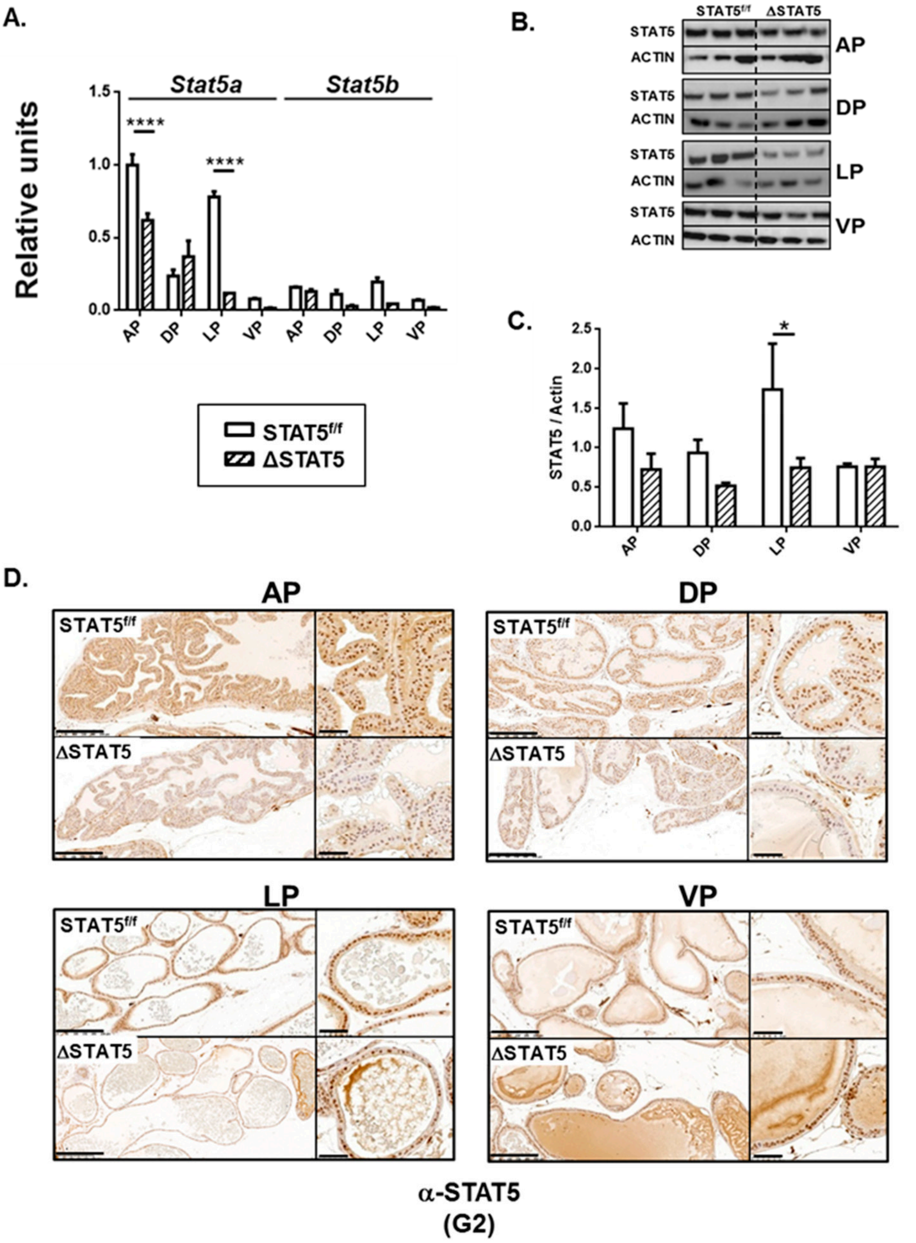

Figure S1. Lobe-specific pattern of STAT5 deletion in 6 month-old △STAT5 mice. **A.** Lobe-specific expression of *STAT5A* and *STAT5B* mRNA in $STAT5^{f/f}$ and △STAT5 mice as determined by RT-qPCR. **B–C.** Lobe-specific expression of STAT5 protein in $STAT5^{f/f}$ and △STAT5 mice as determined by immunoblot (each lane corresponds to a different animal). Quantification of STAT5 expression (STAT5/ACTIN) was performed by densitometry (C). **D**. Immunohistochemical analysis of STAT5 expression in anterior (AP), dorsal (DP), lateral (LP) and ventral (VP) prostates of $STAT5^{f/f}$ and △STAT5 mice, as indicated. *Statistics*: Stars denote significant differences in a repeated-measures two-way ANOVA with Sidak's multiple comparisons. Size bars: 250 μm in large images and 50 μm in insets. * $p < 0.05$; **** $p < 0.0001$.

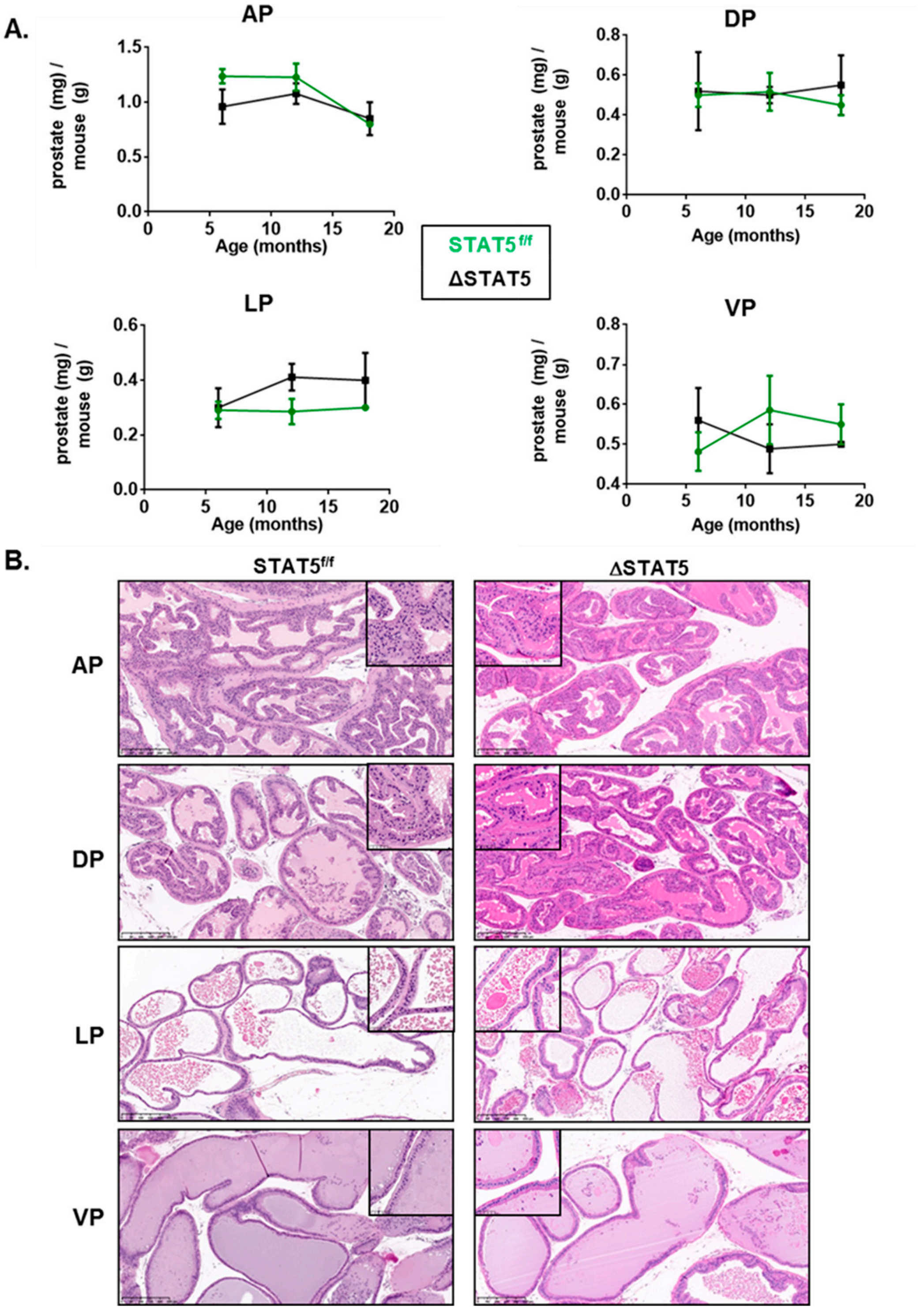

Figure S2. STAT5 deletion has no detectable effect on the prostate tissue. **A.** The prostate weight in 6, 12 and 18 month-old STAT5$^{f/f}$ and ΔSTAT5 mice is expressed as the ratio of total prostate normalized to the weight of corresponding animal. There was no genotype-dependent difference between age-matched mice. **B.** Histological analysis (hematoxylin counterstaining) of the four prostate lobes from 6 month-old STAT5$^{f/f}$ and ΔSTAT5 mice showing no obvious sign of abnormalities. Size bars: 250 μm in large images and 50 μm in insets.

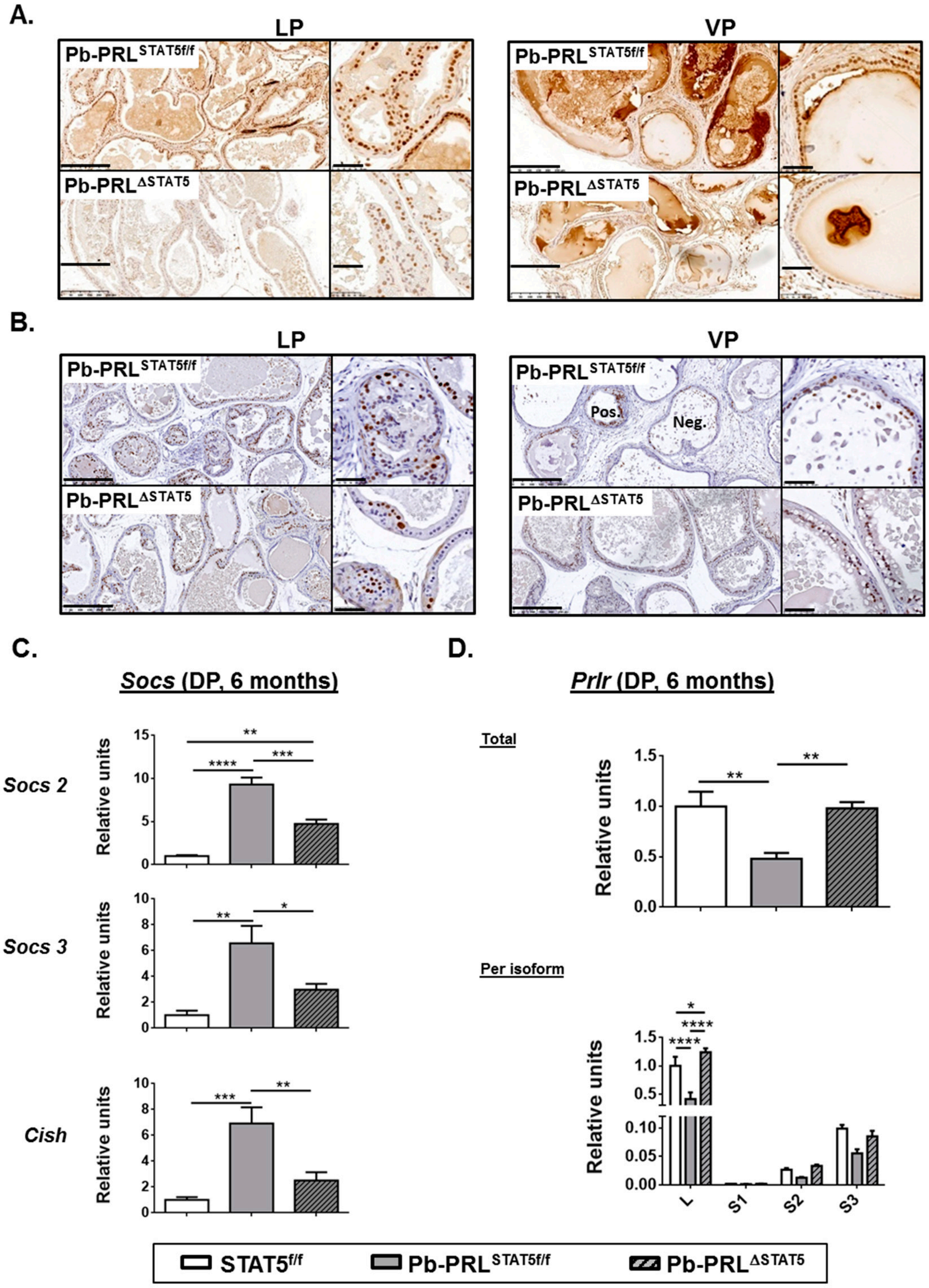

Figure S3. Lobe-specific pattern of STAT5 deletion of 6 month-old Pb-PRL$^{\Delta STAT5}$ mice (complementary to Figure 1). **A–B.** Immunohistochemical analysis of STAT5 expression (A) and phosphorylation (B) in lateral (LP) and ventral (VP) prostates of Pb-PRL$^{STAT5f/f}$ and Pb-PRL$^{\Delta STAT5}$ mice, as indicated (see main Figure 1 for details). **C–D.** The mRNA expression of *SOCS* genes (C) and *PRLR* (D) was monitored in dorsal prostates of 6 month-old STAT5$^{f/f}$, Pb-PRL$^{STAT5f/f}$ and Pb-PRL$^{\Delta STAT5}$ using RT-qPCR. Statistics: Stars denote significant differences in a repeated-measures one-way ANOVA with Tukey's multiple comparisons (C) or two-way ANOVA with Sidak's multiple comparisons (D). Size bars: 250 µm in large images and 50 µm in insets. Size bars: 250 µm in large images and 50 µm in insets. * $p < 0.05$, ** $p < 0.01$, *** $p < 0.001$, **** $p < 0.0001$.

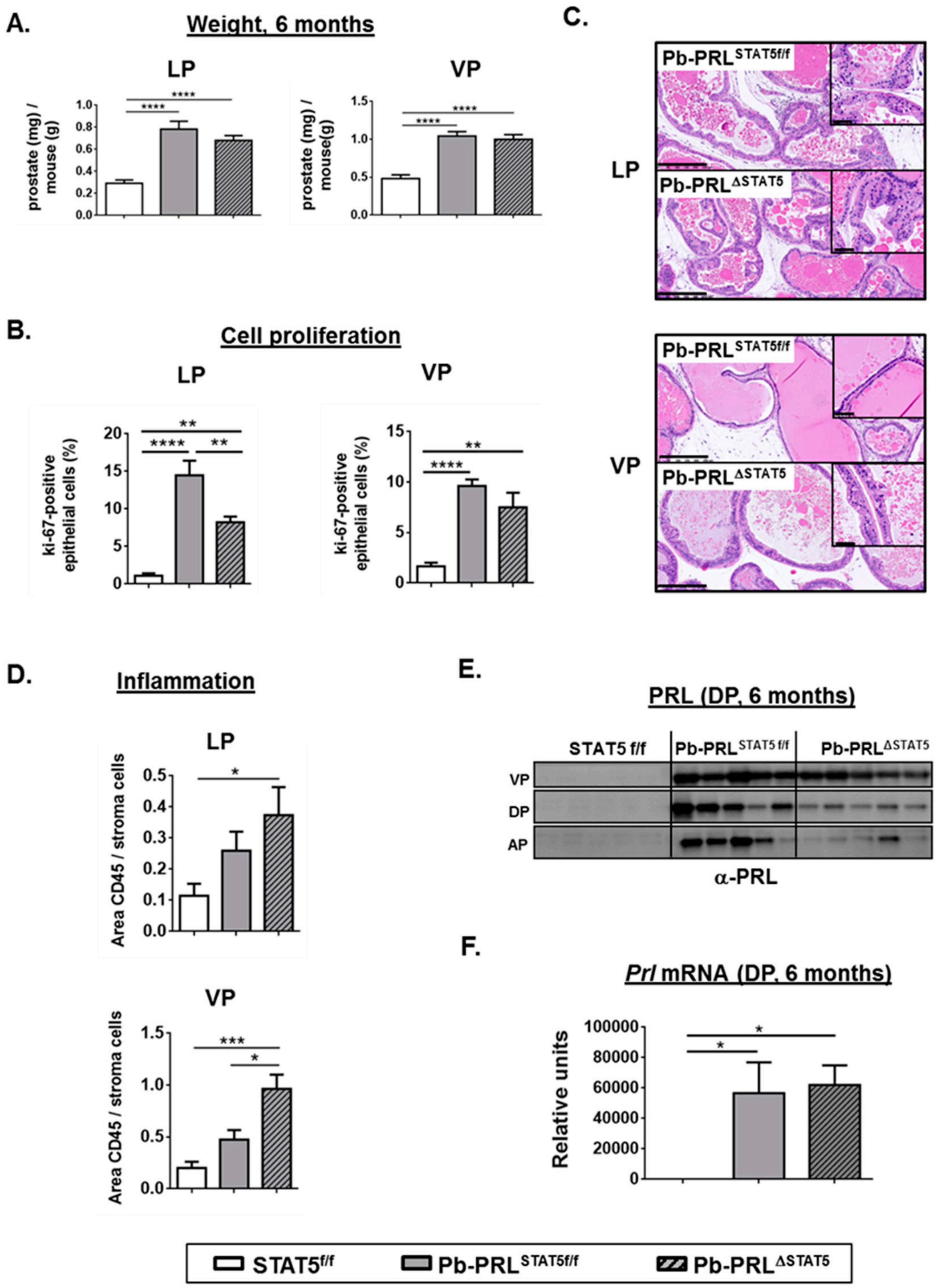

Figure S4. Lobe-specific effects of STAT5 deletion of 6 month-old Pb-PRL$^{\Delta STAT5}$ mice (complementary to Figure 4). **A.** The weight of lateral (LP) and ventral (VP) prostate lobes in 6 month-old STAT5$^{f/f}$, Pb-PRL$^{STAT5f/f}$ and Pb-PRL$^{\Delta STAT5}$ mice is expressed as the ratio of lobe weight normalized to the weight of corresponding animal. **B.** The proliferation index of lateral and ventral prostate was determined in the three genotypes (see Figure 4 for details). **C.** Histological analysis (hematoxylin counterstaining) of lateral and ventral lobes of Pb-PRL$^{STAT5f/f}$ and Pb-PRL$^{\Delta STAT5}$ mice showing similar hyperplasia in both genotypes. **D.** Inflammation was identified using CD45 immunostaining. The degree of inflammation was quantified using Calopix software and is represented as the ratio of CD45+ area *versus* stroma area. **E,F.** Expression of rat *PRL* transgene was monitored using immunoblot (D) and RT-qPCR (E) in the three genotypes. Stars denote significant differences in a repeated-measures one-way ANOVA with Tukey's multiple comparisons. * $p < 0.05$, ** $p < 0.01$, *** $p < 0.001$, **** $p < 0.0001$.

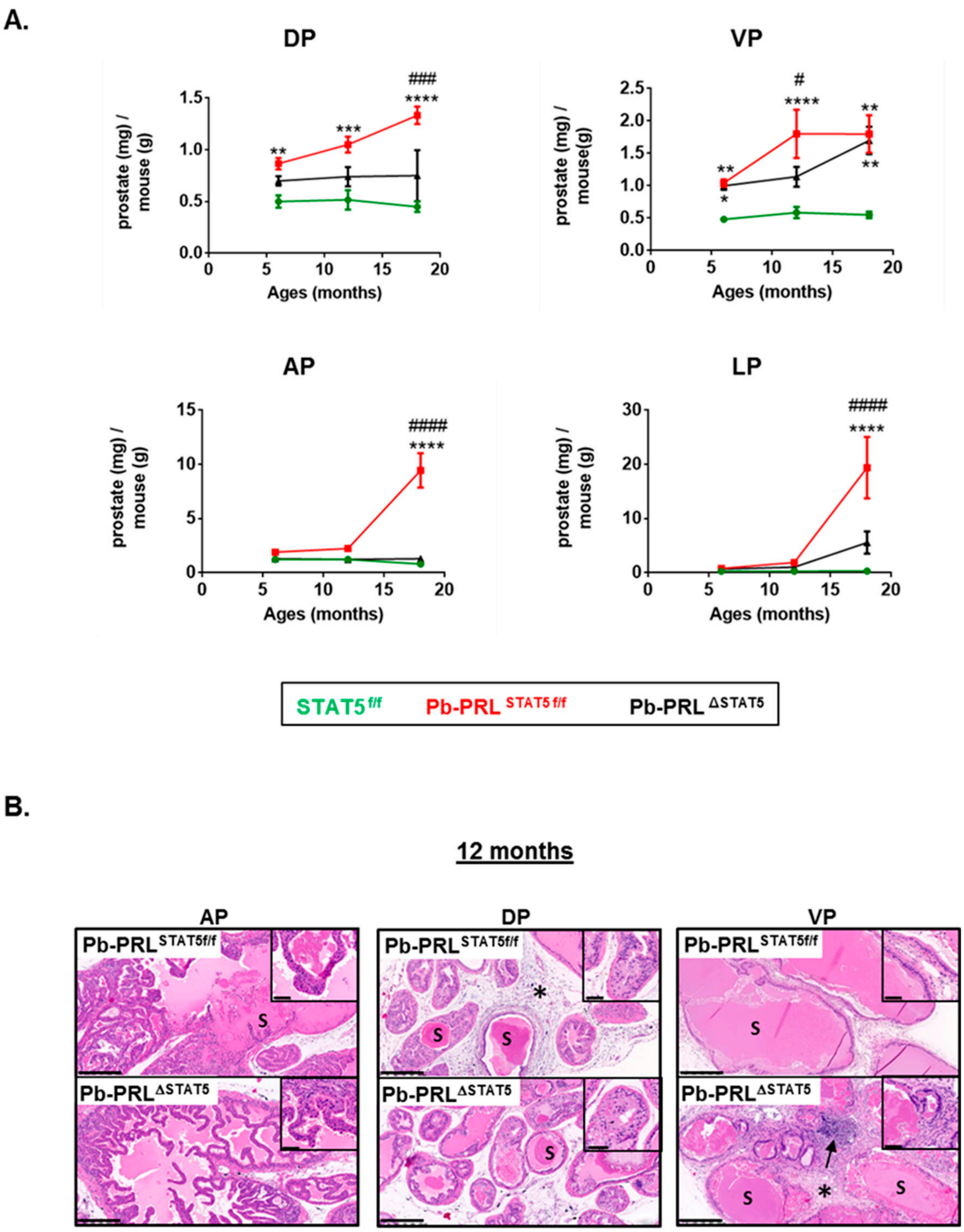

Figure S5. STAT5 deletion does not prevent prostate tumor progression in aged Pb-PRL mice (complementary to Figure 5). **A.** The prostate weight in 6, 12 and 18 month-old STAT5$^{f/f}$, Pb-PRL$^{STAT5f/f}$ and Pb-PRL$^{\Delta STAT5}$ mice is expressed as the ratio of total prostate weight normalized to the weight of corresponding animal. Symbols: * $p < 0.05$, ** $p < 0.01$, *** $p < 0.001$, **** $p < 0.0001$ (versus STAT5$^{f/f}$); # $p < 0.05$, ## $p < 0.01$, ### $p < 0.001$, #### $p < 0.0001$ (versus Pb-PRL$^{\Delta STAT5}$). **B.** Histological analysis (hematoxylin counterstaining) of the anterior (AP), dorsal (DP) and ventral (VP) prostate lobes from 12 month-old Pb-PRL$^{STAT5f/f}$ and Pb-PRL$^{\Delta STAT5}$ mice showing similar abnormalities including PINs, increased stromal density (stars), inflammation (arrows) and dense eosinophilic secretions (S). Statistics: Symbols (*: vs age-matched STAT5$^{f/f}$; #: versus age-matched Pb-PRL$^{\Delta STAT5}$) denote significant differences in a repeated-measures two-way ANOVA with Tukey's multiple comparisons. Size bars: 250 µm in large images and 50 µm in insets.

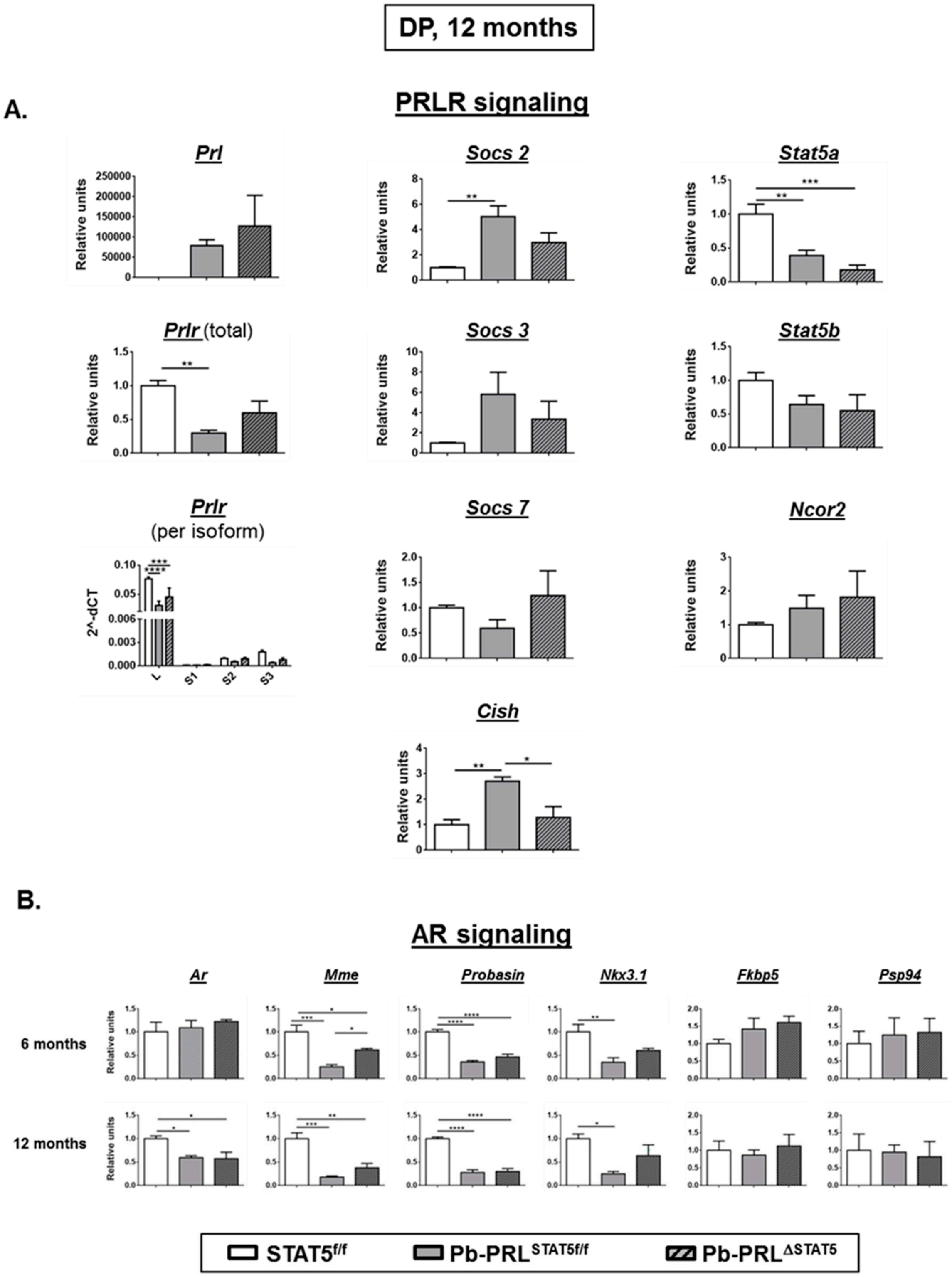

Figure S6. Characterization of PRLR and AR signaling pathways in dorsal prostate of 12 month-old STAT5$^{f/f}$, Pb-PRL$^{STAT5f/f}$ and Pb-PRL$^{\Delta STAT5}$ mice (complementary to Figure 6). **A.** The expression of various actors of the PRLR pathway as determined by RT-qPCR was analyzed in dorsal prostates of 12 month-old STAT5$^{f/f}$, Pb-PRL$^{STAT5f/f}$ and Pb-PRL$^{\Delta STAT5}$ mice. **B.** The expression of AR and of target genes of the AR pathway as determined by RT-qPCR was analyzed in dorsal prostates of 6 and 12 month-old STAT5$^{f/f}$, Pb-PRL$^{STAT5f/f}$ and Pb-PRL$^{\Delta STAT5}$ mice, as indicated. In both panels data are expressed as fold-induction versus STAT5$^{f/f}$ samples. Statistics: Stars denote significant differences in a repeated-measures one-way ANOVA with Tukey's multiple comparisons. Except for PRLR (per isoform), stars denote significant differences in a repeated-measures two-way ANOVA with Tukey's multiple comparisons. * $p < 0.05$, ** $p < 0.01$, *** $p < 0.001$, **** $p < 0.0001$.

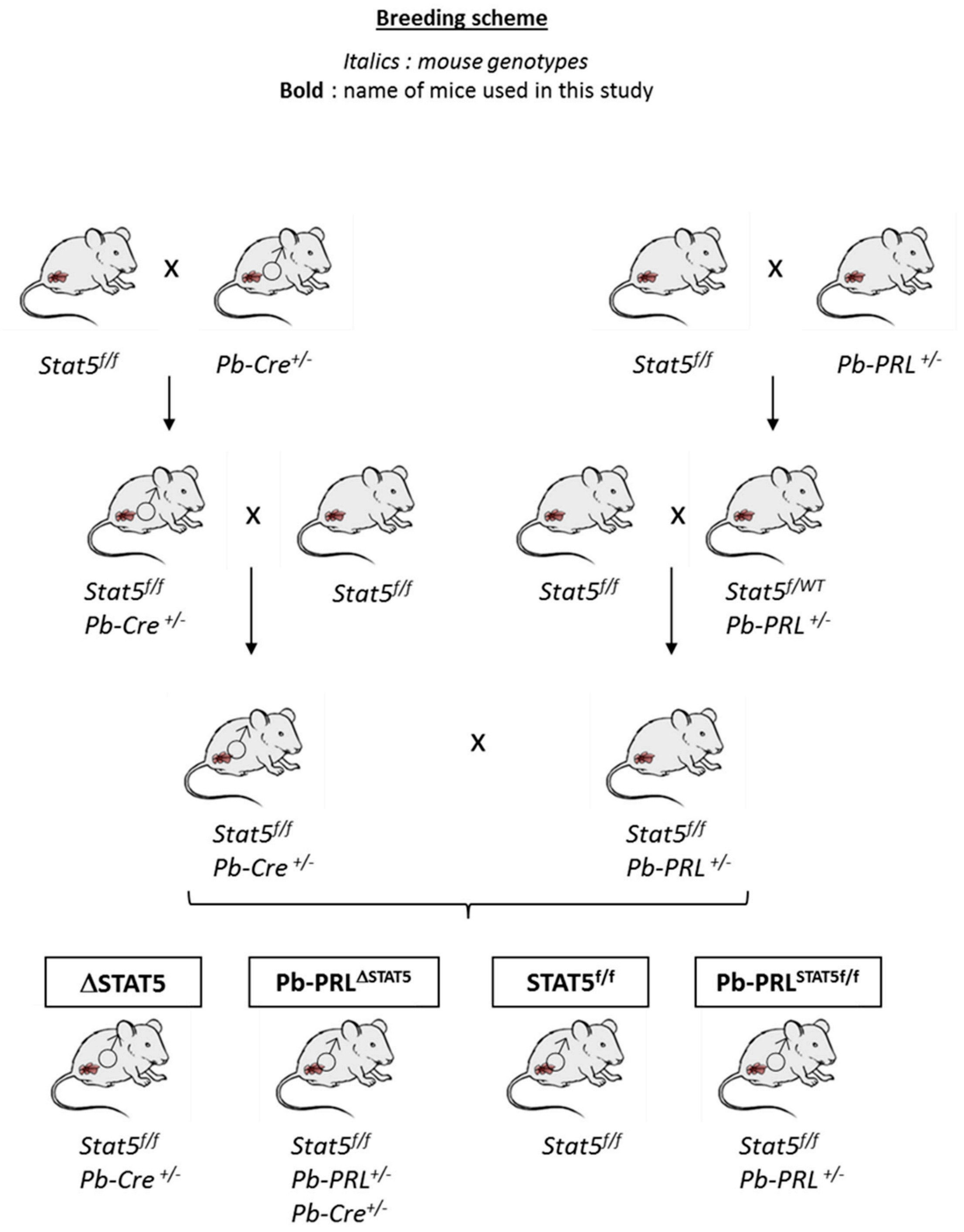

Figure S7. Breeding scheme.

Western-blots corresponding to Fig. 1B

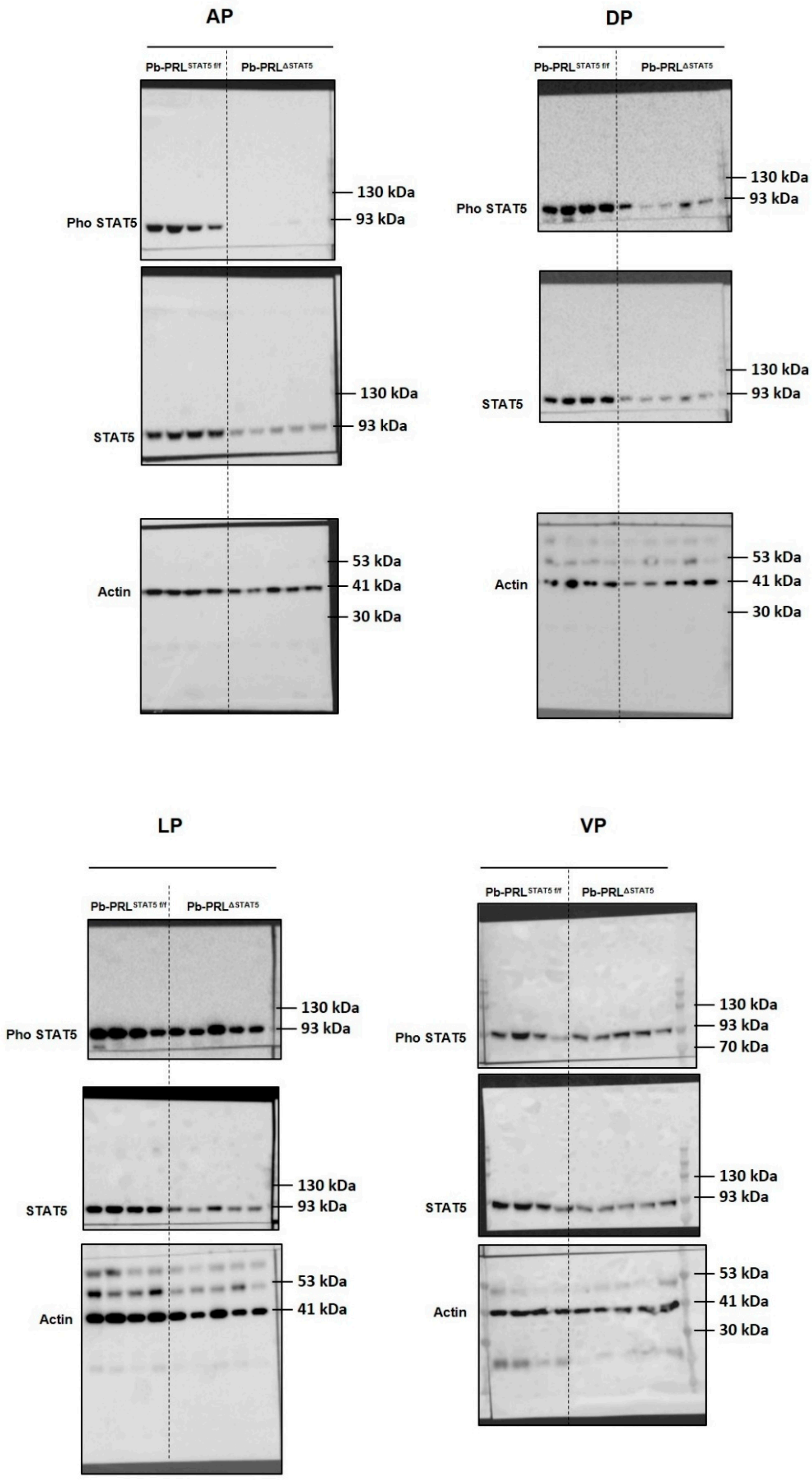

Figure S8. *Cont.*

Western-blots corresponding to Fig. 4A

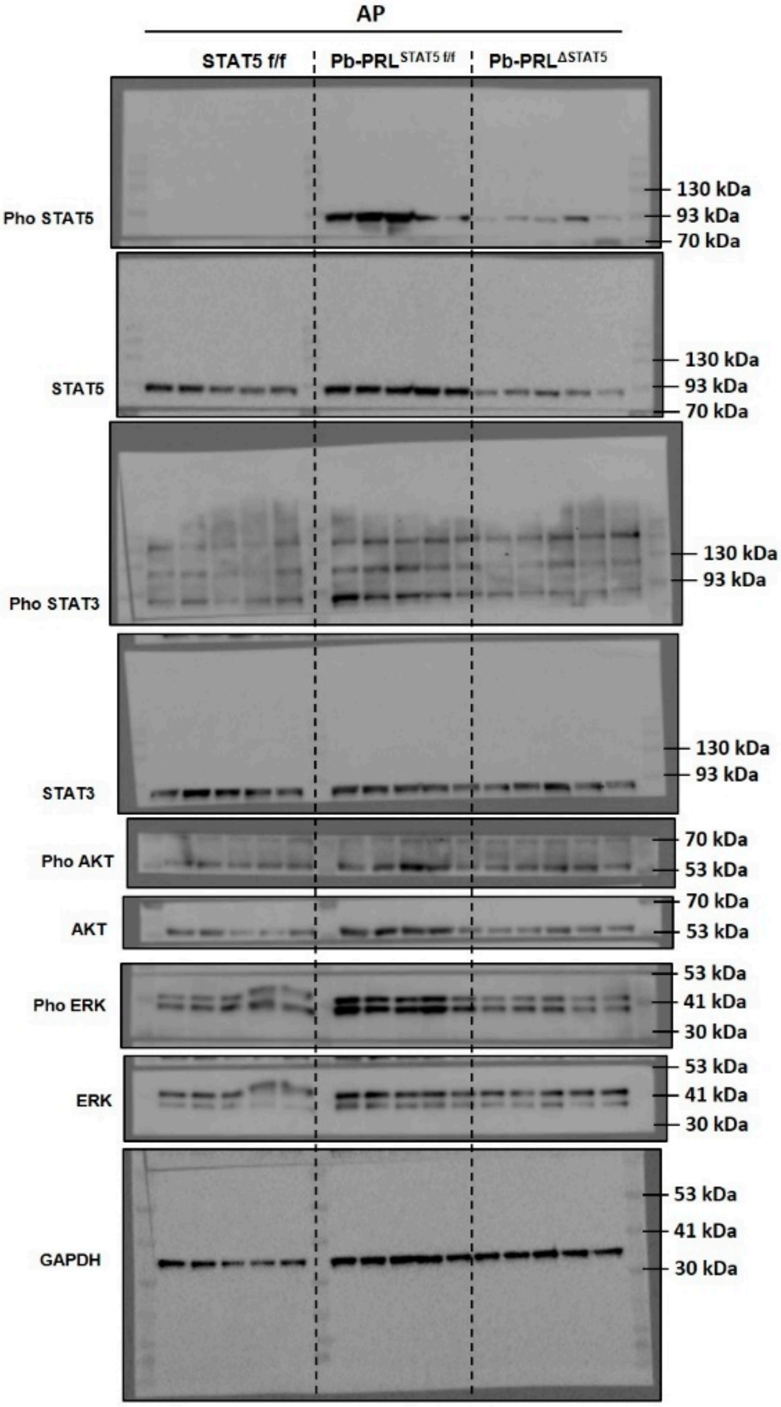

Figure S8. *Cont.*

Western-blots corresponding to Fig. 4B

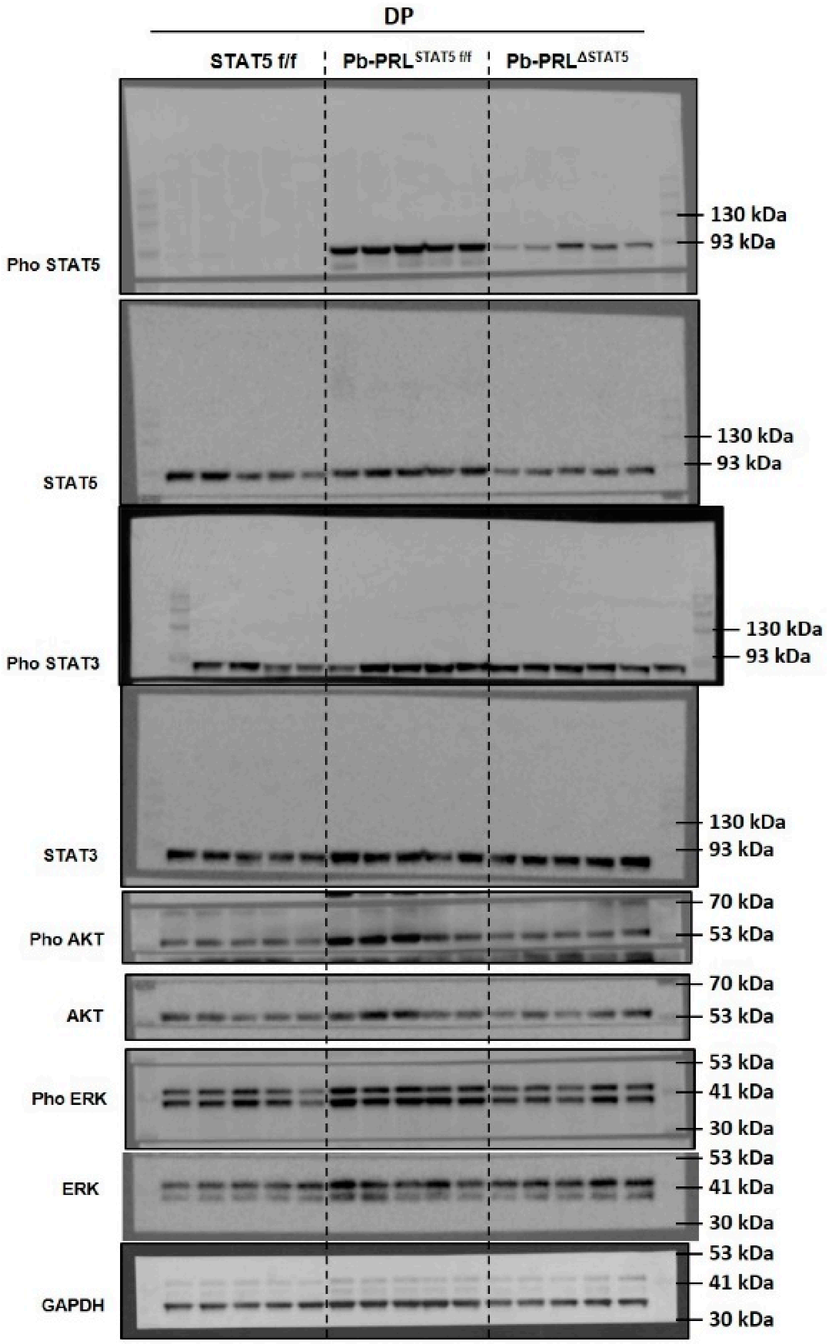

Figure S8. *Cont.*

Western-blots corresponding to Fig. 4C

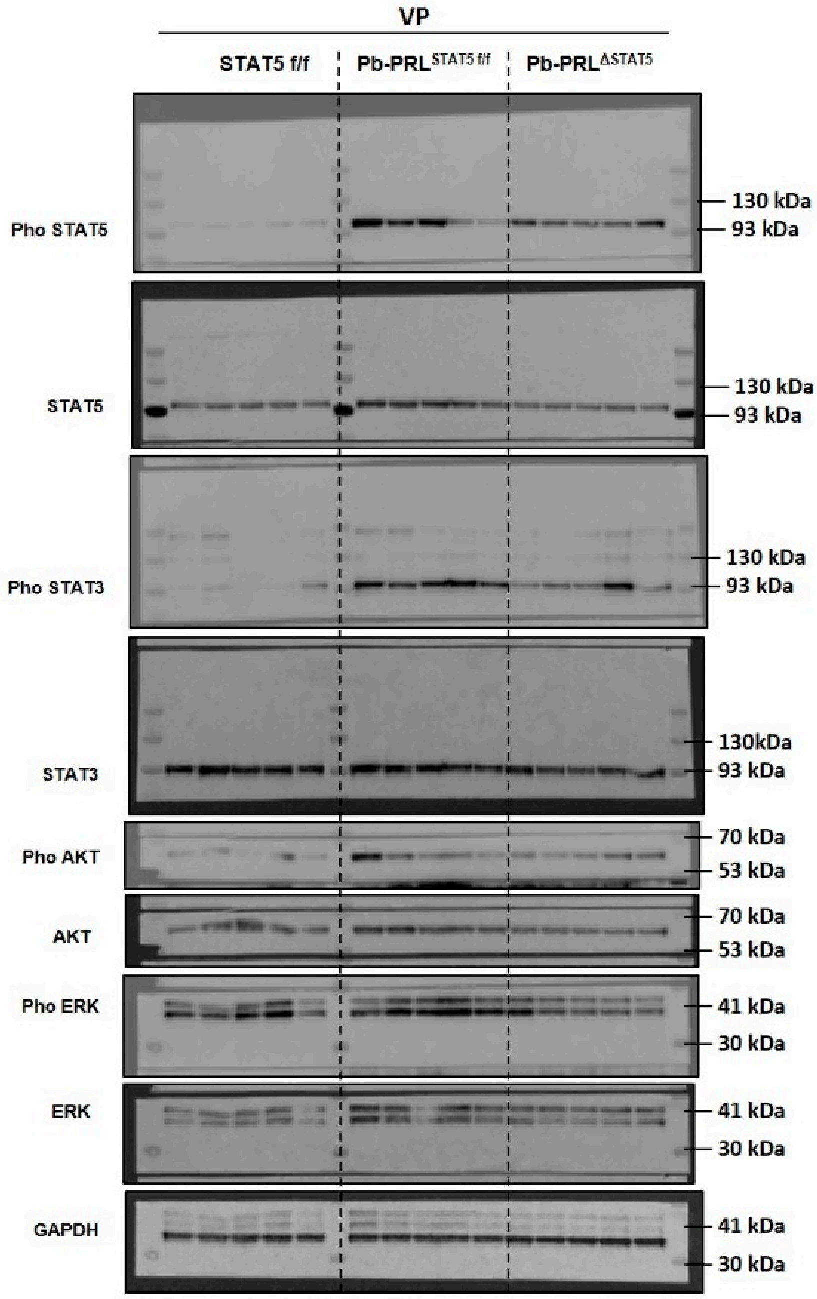

Figure S8. *Cont.*

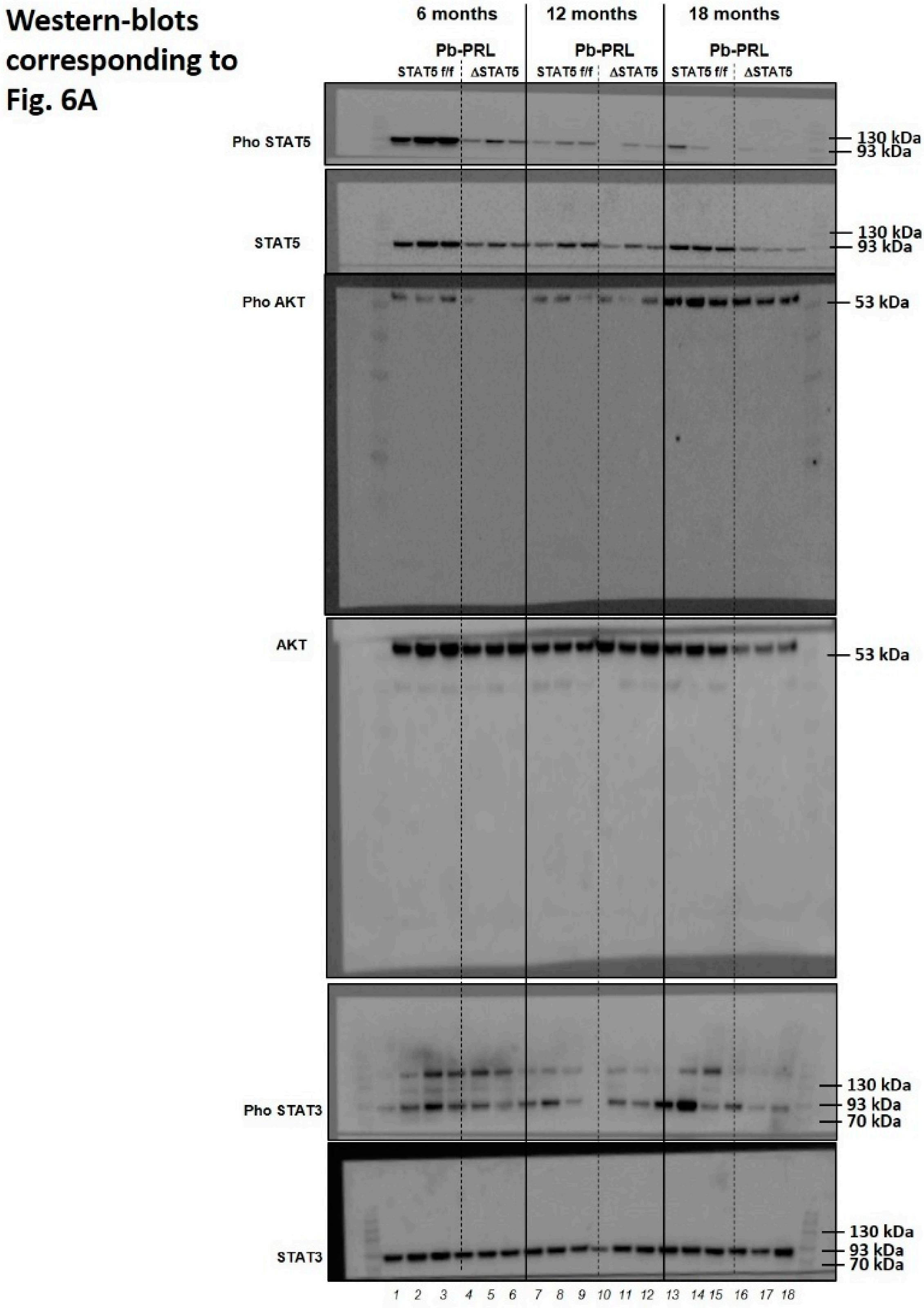

Figure S8. *Cont.*

Western-blots corresponding to Fig. 6A (continued)

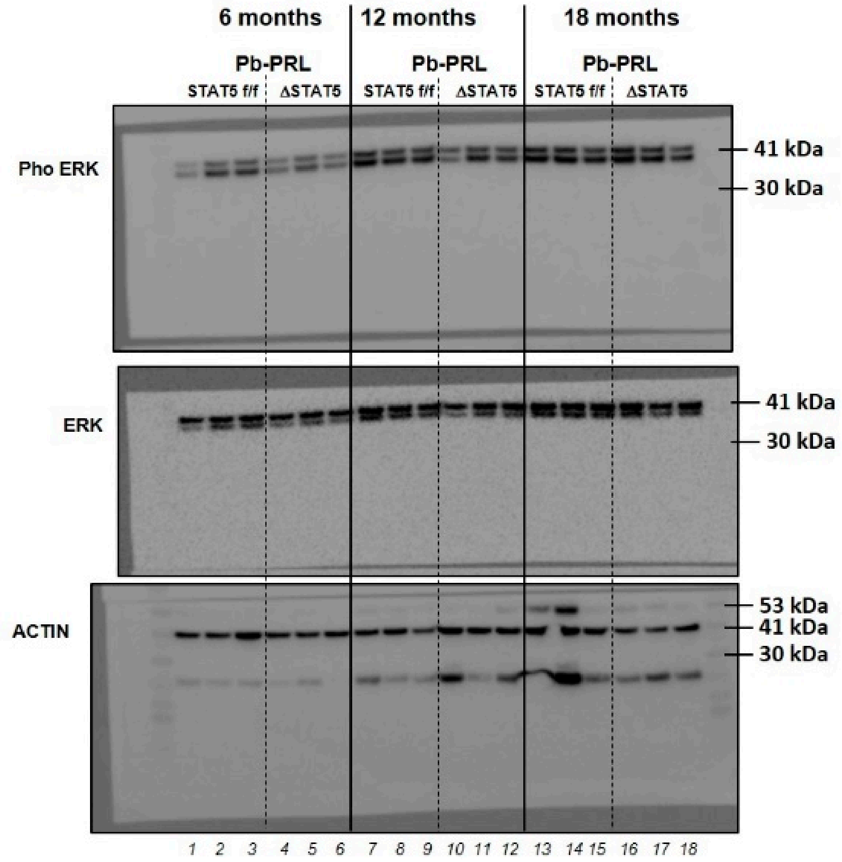

Figure S8. *Cont.*

Western-blots corresponding to Fig. S1B

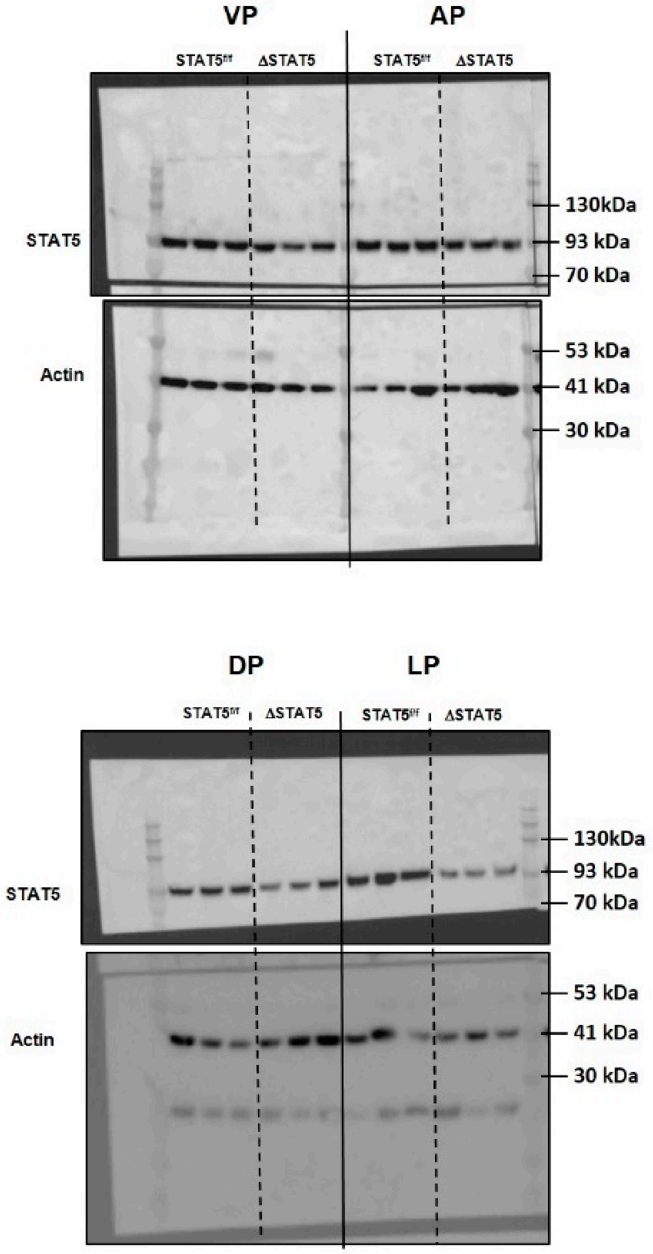

Figure S8. *Cont.*

Western-blots corresponding to Fig. S4E

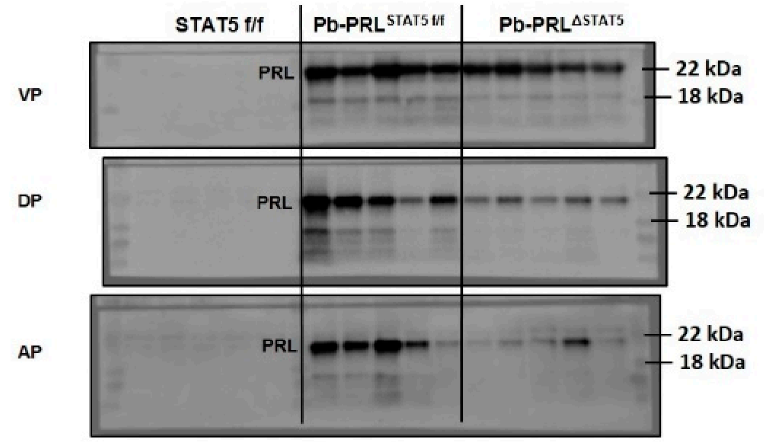

Figure S8. Whole Western blot membranes corresponding to representative images shown in main and supplemental Figures, as indicated (continued on the next pages)

Table S1. Antibody references and conditions of use.

Target	Ref./Clone	Provider	Dilution
**STAT5**	Sc 835/C-17	Santa cruz	Blot: 1/2,000
**STAT5b**	Sc 1656/G2 *	Santa cruz	IHC: 1/200
**pSTAT5**	9359/C11C5	Cell Signaling	Blot: 1/1000<br>IHC: 1/300
**STAT3**	12640/D3Z2G	Cell Signaling	Blot: 1/1000
**pSTAT3**	9131S	Cell Signaling	Blot: 1/1000
**Erk1/2**	9102	Cell Signaling	Blot: 1/3000
**pErk1/2**	4370/D13.14.4E	Cell Signaling	Blot: 1/1000
**Akt**	9272	Cell Signaling	Blot: 1/1000
**pAkt**	9271S	Cell Signaling	Blot: 1/1000
**CD45**	sc-53665/30-F11	Santa Cruz	IHC: 1/150
**Ki-67**	RM9106/SP6	Thermo Scientific	IHC: 1/300
**GAPDH**	2118S/14C10	Cell Signaling	Blot: 1/1000
**Actin**	A5316	Sigma	Blot: 1/5000
**PRL**	Anti-mPRL-IC<br>AFP879151	National hormone & peptide program (Dr. Parlow)	Blot: 1/1000

* maps amino acids 750-779 that are common to both STAT5 isoforms.

Table S2. Primers used for qPCR.

Gene	Name	5′-3′ sequence
Cyclophilin	Cyclophylin A-R	TTGCTGGTCTTGCCATTCCT
	Cyclophylin A-R	CAGGTCCTGGCATCTTGTCC
Probasin	Mouse F	GCATGTGCTAGGCGTCTCC
	Mouse R	GTTCTCAATGGTGAGCCTTCAT
Stat5a	Mouse F	CATTGCTTGGAAGTTTGACTCTC
	Mouse R	CACGTAGATAAGGTAGTTCAGGTC
Stat5b	Mouse F	GCACCTTCAGATCAACCAAAC
	Mouse R	CAGCTGGGCAAACTGAG
Socs 1	Mouse F	ATTCCACTCCTACCTCTCCAT
	Mouse R	CAGAAAAATGAAGCCAGAGACC
Socs 2	Mouse F	GCGCGAGCTCAGTCAAAC
	Mouse R	CTGGAGCCTCTTTTAATTTCTCTTT
Socs3	Mouse F	GAGATTTCGCTTCGGGACTA
	Mouse R	GGAAACTTGCTGTGGGTGA
Cish	Mouse F	GCTCCTTTCTCCTTATCC
	Mouse R	CCGCCAATTTGCTCCA
Ncor2	Mouse F	TGGAACCCGGCACGC
	Mouse R	GGTAGGGTAGACCCCTT
Prlr *(all isoforms)*	Mouse F	ATAAAAGGATTTGATACTCATCTGCTAGAG
Prlr L	Mouse R	TGTCATCCACTTCCAAGAACTCC
Prlr S1	Mouse R	CATAAAAACTCAGTTGTTGGAATCTTCA
Prlr S2	Mouse R	GGAAAAAGACATGGCAGAAACC
Prlr S3	Mouse R	AGTTCCCCTTCATTGTCCAGTTT
Ar	Mouse F	GAGAGAGGCAGCTTGTGCAT
	Mouse R	TACTGAATGACCGCCATCTG
Nkx3.1	Mouse F	ATGCTTAGGGTAGCGGAGC
	Mouse R	TGCGGATTGCCTGAGTGTC
Fkbp5	Mouse F	TTTGAAGATTCAGGCGTTATCCG
	Mouse R	GGTGGACTTTTACCGTTGCTC
Mme	Mouse F	GGTTGAAACGCAACGTCATTC
	Mouse R	CTGCACTGCTACTATGTCTTCAG
Psp94	Mouse F	TGGTGATAGCATCCAAAGCA
	Mouse R	GCTTGTTACCATCAGCATCC
Probasin	Mouse F	GCATGTGCTAGGCGTCTCC
	Mouse R	GTTCTCAATGGTGAGCCTTCAT

© 2019 by the authors. Licensee MDPI, Basel, Switzerland. This article is an open access article distributed under the terms and conditions of the Creative Commons Attribution (CC BY) license (http://creativecommons.org/licenses/by/4.0/).
